# A Review of RedOx Cycling of Solid Oxide Fuel Cells Anode

**DOI:** 10.3390/membranes2030585

**Published:** 2012-08-31

**Authors:** Antonin Faes, Aïcha Hessler-Wyser, Amédée Zryd, Jan Van Herle

**Affiliations:** 1 Design & Materials Unit (UDM), University of Applied Sciences Western Switzerland (HES-SO Valais), Sion 1950, Switzerland; Email: amedee.zryd@hevs.ch; 2 Interdisciplinary Centre for Electron Microscopy (CIME), Ecole Polytechnique Fédérale de Lausanne (EPFL), Lausanne 1015, Switzerland; Email: aicha.hessler@epfl.ch; 3 Industrial Energy Systems Laboratory (LENI), EPFL, Lausanne 1015, Switzerland; Email: jan.vanherle@epfl.ch

**Keywords:** SOFC anode, RedOx cycle, nickel reduction and oxidation, reoxidation instability, solid oxide fuel cell durability.

## Abstract

Solid oxide fuel cells are able to convert fuels, including hydrocarbons, to electricity with an unbeatable efficiency even for small systems. One of the main limitations for long-term utilization is the reduction-oxidation cycling (RedOx cycles) of the nickel-based anodes. This paper will review the effects and parameters influencing RedOx cycles of the Ni-ceramic anode. Second, solutions for RedOx instability are reviewed in the patent and open scientific literature. The solutions are described from the point of view of the system, stack design, cell design, new materials and microstructure optimization. Finally, a brief synthesis on RedOx cycling of Ni-based anode supports for standard and optimized microstructures is depicted.

## Content

1. **Introduction**
2. **RedOx instability**
  2.1. *Problematic*
  2.2. *High Temperature Nickel Oxide Reduction and Nickel Oxidation*
    2.2.1. Reduction of NiO
    2.2.2. High Temperature Oxidation of Ni
  2.3. *Reduction of NiO-YSZ Cermet*
  2.4. *Oxidation of Ni-Ceramics Composite*
    2.4.1. Kinetics of Oxidation
    2.4.2. Homogeneous Versus Inhomogeneous Oxidation
    2.4.3. Expansion during Reoxidation
    2.4.4. Bending and Stresses in Half-Cell Samples (Anode Support)
    2.4.5. Young’s Modulus and Strength Variation with Reoxidation
    2.4.6. RedOx Expansion Limits: Mathematical Approaches
    2.4.7. Electrical Conductivity versus RedOx Cycles
    2.4.8. Temperature Variation during Oxidation
    2.4.9. Reoxidation by Ionic Current
    2.4.10. Micro and Nano-Structural Changes upon Redox Cycling
    2.4.11. Electrochemical Performance and Electrochemical Impedance Spectroscopy
    2.4.12. Single Chamber SOFC
  2.5. *Summary of the RedOx Instability*
3. **RedOx Solutions**
  3.1. *System Solutions*
    3.1.1. Dependent System Solutions
    3.1.2. Passive System Solutions
    3.1.3. Active System Solutions
  3.2. *Stack Design*
    3.2.1. Planar Design
    3.2.2. Tubular Design
  3.3. *Cell Design*
    3.3.1. Cathode Supported Cell (CSC)
    3.3.2. Electrolyte Supported Cell (ESC)
    3.3.3. Metal Supported Cell (MSC)
    3.3.4. Inert Substrate Supported Cells (ISSC)
    3.3.5. Anode Supported Cell (ASC)
  3.4. *Modification of the Microstructure*
    3.4.1. Anode Functional Layer, Anode Support and Anode Current Collecting Layer
    3.4.2. Particles Size
    3.4.3. Sintering Temperature
    3.4.4. Porosity
    3.4.5. Composition
    3.4.6. Orientation and Particle Shape of Nickel Phase
    3.4.7. Ni coated Pore-Former
    3.4.8. Ni Foam
    3.4.9. Wet Impregnation (WI)
    3.4.10. Ni Coated Ceramic
    3.4.11. Graded Composition and Porosity
    3.4.12. Controlled RedOx Cycle
  3.5. *Alternative Anode Materials*
    3.5.1. Alloys and Additives for Metal-Ceramic Anode
    3.5.2. Full Ceramic Anode
    3.5.3. Mechanically Stronger Materials
    3.5.4. Use Support with Higher Thermal Expansion Coefficient (TEC)
  3.6. *Kinetics*
    3.6.1. Oxidation Barrier
    3.6.2. Improved Sealing
    3.6.3. Lower Operating Temperature
4. **Synthesis for Ni-Based Anode-Supported Cells**
5. **Conclusions**
6. **Acknowledgments**
7. **Appendix**
8. **References**

## 1. Introduction

Fuel cells will play a key role in the future as (1) they convert fuel to electricity with high efficiency (>60%) even for small systems; (2) in electrolyzer mode, they can produce hydrogen from electricity and water to store energy; (3) they are clean (negligible NO_x_ and SO_x_ emissions) and (4) they are silent.

Solid oxide fuel cells (SOFCs) are based on a ceramic electrolyte and work at elevated temperature between 600 and 1000 °C. Their advantages are (1) design flexibility thanks to the solid electrolyte; (2) fuel flexibility including hydrogen, hydrocarbons and bio-fuels and (3) co-generation of heat and electricity (reaching total efficiencies up to 95%) [1].

Patented in the 1970s, the state-of-the art anode is based on a nickel-ceramic composite material due to its high activity, electrical conductivity and relatively low cost [2]. The goals of the ionically conducting ceramic are, first, to limit nickel agglomeration at high temperature, second to increase the active electrode thickness and finally to match the anode thermal expansion coefficient (TEC) to that of the ceramic electrolyte. The most used ceramics are yttria stabilized zirconia (YSZ) and gadolinia doped ceria (GDC). During its fabrication, the anode is sintered at elevated temperature (1300–1450 °C), producing a NiO-ceramic composite. The first anode utilization reduces the nickel oxide and creates a porous structure due to the volume reduction from NiO to Ni. 

Majors limitations of the Ni-ceramic anodes are (1) the nickel microstructural changes to lower the interfacial energy, which decreases the electrochemical activity [3,4,5]; (2) the volatilization of the nickel under high steam concentration [6,7]; (3) promotion of the competitive catalytic cracking of hydrocarbons that produces a rapid deposition of carbon in the anode [8]; (4) impurities in the fuel stream, particularly sulfur and phosphorus, that inhibit anode functionality [9] and (5) anode expansion during re-oxidation of the Ni if a fuel supply cut occurs, under high fuel utilization operation or with seal leakage occurrence [10,11,12,13]. 

Nickel is not stable, at high temperature, against oxidation in air. The volume changes during successive reduction and reoxidation cycles (“RedOx cycle”) may be detrimental for the anode unity. The problem is even worse if the cell design is anode supported because the volume change puts the electrolyte under tension and, once cracked, produces leakage between fuel and oxidant gases [10].

During the last 15 years, a large amount of work has been carried out on the RedOx problematics of SOFC anodes, including three review papers (two considering Ni-YSZ anodes [12,14] and one focused on ceramic anodes [15]), about 10 PhD theses [16,17,18,19,20,21,22,23,24], tens of patents and hundreds of scientific papers. This review will try to be as exhaustive as possible. It describes first the reduction and oxidation of nickel at high temperature, then continues with the reduction and oxidation of Ni-based composites. The effects of RedOx cycles on ceramic-metal (cermet) anode properties, like conductivity, electrochemical performance, dimension, *etc.*, are reported. Finally, it reviews the solutions at system, design and materials levels. A brief synthesis precedes the conclusion. 

## 2. RedOx Instability

### 2.1. Problematic

RedOx instability refers to the chemo-mechanical instability of the solid oxide fuel cell anode and support under oxygen partial pressure variation of more than 20 orders of magnitude during reduction and oxidation (*p_O2,air_* = 0.21 atm and *p_H2,3%H2O,800°C_* = 4 × 10^−22^ atm) at high temperature (600–1000 °C). 

This was first reported in 1996 by Cassidy *et al.* for Ni-YSZ anode supported thin electrolyte cells [10]. The volume increase upon reduction and reoxidation (“RedOx cycle”) of the anode support was measured as well as the loss of the open circuit voltage (OCV) due to cracking of the thin electrolyte. 

**Figure 1:**
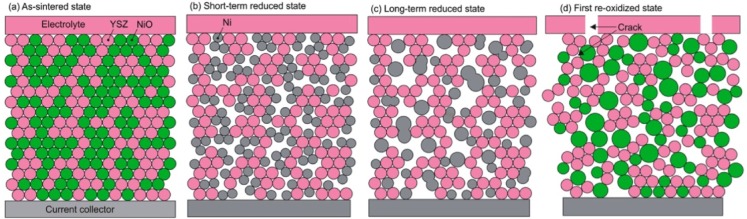
Microstructural changes during a RedOx process in Ni-YSZ (yttria stabilized zirconia) based anodes [27].

This pointed out one of the main limitations of the nickel-ceramic based anode. These anodes show a large bulk volume change upon Ni reoxidation. The shrinkage of nickel oxide particles during reduction is around 40 vol %, and during reoxidation nickel expansion is around 66 vol %. The molar volumes of NiO and Ni are given in Table 1. The ratio of molar volume of the oxide and the metal is known as the Pilling–Bedworth ratio and is about 1.66 for nickel [25]. Based on Cassidy’s and following works, Klemensø drew a schematic of the mechanisms underlying the anode RedOx, as shown in Figure 1 [26,27,28].

**Table 1:** Nickel and nickel oxide molar mass, specific mass and molar volume [29,30].

	NiO	Ni
**M [g/mol]**	74.71	58.71
***ρ* [g/cm^3^]**	6.67	8.9
**V [cm^3^/mol]**	10.97	6.58

Anode reduction increases porosity because of the NiO to Ni volume change. During utilization, the metallic nickel phase re-organizes due to high temperature, water vapor content and surface tension equilibrium [3,31,32]. If the oxygen partial pressure increases, nickel can rapidly oxidize at high temperature (above 600 °C). The ensuing volume increase can then destroy the electrolyte and the anode support.

Reoxidation of Ni can occur for a variety of reasons at the operating temperature:

- Under high load or high fuel utilization conditions, the oxygen partial pressure can locally increase up to a critical value [33];
- The oxygen partial pressure increases in the vicinity of compressive seals, which causes small air leakage to the anode [34];
- Accidental fuel supply interruption;
- To reduce cost and system complexity, shut down and start up is done without protective gas.

This limitation of the state-of-the-art Ni-YSZ anode induced a large research effort from the scientific community as it is considered as one of the bottlenecks of SOFC technology [35]. Before considering the composite, the reduction and oxidation of pure nickel is discussed.

### 2.2. High Temperature Nickel Oxide Reduction and Nickel Oxidation

#### 2.2.1. Reduction of NiO

The reduction of NiO occurs by H_2_ supply and H_2_O removal according to Equation (1). The kinetics of NiO reduction in H_2 _are commonly approximated by a linear equation with time at constant temperature (Equation (2)), implying a surface controlled process [36]. Usually the slope is taken at a certain conversion degree (*x* between 20% and 80%) and its logarithm reported against T^−1^ to obtain an activation energy (*E_a_*), as the reaction is thermally activated and follows an Arrhenius law (Equation (3)). Deviation of linear kinetics at low conversion degree is due to an initial induction period for nucleation of Ni clusters, which then grow at a linear rate. At the end of the reaction, it slows down as the diffusion path for H_2_ reactant and H_2_O product gets longer through the porous Ni metallic layer. This will thus give an “s”-shape curve at low temperature; at high temperature, the induction period is short and the densification of Ni at the surface decreases the gas diffusion process further. This is a reason for lower activation energy reported at higher temperature (see Table 2). Richardson *et al.* presented a good description of NiO reduction by hydrogen [37]. More generally, there are multiple reaction rate equations describing the reduction of metals like a power law, Avrami kinetics or first order kinetics [37,38].
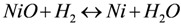
(1)

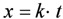
(2)

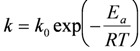
(3)
with *x* the degree of conversion, *k* the reaction rate, *t* the time, *k*_0_ the reaction rate constant, *E_a_* the activation energy, *R* the gas constant (8.314 J mol^−1^ K^−1^) and *T* the temperature.

**Table 2:** Reduction kinetics for NiO with H_2_ from Richardson *et al.* [37] and other authors.

Source reference	Sample and measurement technique	Temperature range (°C)	E_a_ (kJ mol^−1^)
**Pure nickel oxide**			
Szekely and Evans [37]	Large porous single pellet, TGA	372–753	17
Deb Roy and Abraham [37]	Non-porous spherical pellets, TGA	400–800	22
Bandrowski *et al.* [37]	Large porous NiO pellet in a packed bed, H_2_O detection	261–298	52
Szekely and Evans [37]	Large porous NiO pellet, TGA	226–308	65
Nakajima *et al.* [37]	Powdered NiO sample	277–377	69
Rao and Rashed [39]	Thin NiO slab, TGA	300–400	73
Richardson *et al.* [37]	Porous NiO powder, TGA	220–355	84
Richardson *et al.* [37]	Porous NiO powder thin slab, XRD	175–300	85
Szekely *et al.* [37]	Pressed thin discs, TGA	224–259	133
**NiO and ceramic composite**			
Modena *et al.* [40]	Tape-cast NiO-YSZ, TGA	700–800	25–29
Modena *et al.* [40]	Tape-cast NiO-YSZ 2nd reduction, TGA	700–800	51–87
Waldbillig *et al.* [41]	Tape-cast NiO-YSZ, TGA	500–950	54–78
Tikekar *et al*. [36]	Pressed NiO-YSZ rectangular bar, thickness of reduced layer	600–800	94
Pihlatie *et al*. [38]	Tape-cast NiO-YSZ, TGA	500–750	84

Both nickel and its oxide have a face-centered cubic (FCC) structure with the respective lattice parameters equal to 0.368 and 0.418 nm. Nickel growth is epitaxial on NiO even if the difference in lattice parameter is 13.6% [42,43]. The reduction rate is fairly high: at 600 °C a 0.5 mm NiO particle is reduced in 30 min (32% H_2_ in N_2_). At higher temperature, the kinetics become distorted by sintering of the porous Ni, which limits the access of gas to the oxide [44]. Addition of water vapor to hydrogen reduces the reduction rate and increases the activation energy at low temperature 175–300 °C for relatively coarse particles (10–20 µm) (for 20% H_2_ in N_2_) [37]. Contradictorily, Müller relates that if the water vapor is increased from 3% to 10%, the reduction temperature decreases and the rate increases for fine NiO particles of 0.5 µm (for 6% H_2_ in N_2_) [16]. 

#### 2.2.2. High Temperature Oxidation of Ni

This section is based on three different books [25,45,46] and a review paper from Atkinson [47] describing high temperature oxidation of metals. 

The oxidation forms on top of the metal an oxide layer that separates the gas containing the oxidant species and the metal. In case of nickel, the reaction occurring in air is Ni + ½O_2_ = NiO. For a thin oxide layer (< about 0.1 µm), transport is governed by the electric field built within the layer by the cathodic reaction (½O_2 _+ 2e^−^ = O^2−^) and the anodic one (Ni = Ni^2+ ^+ 2e^−^). This oxidation rate is described by the Mott–Cabrera theory and follows logarithmic laws. 

**Figure 2:**
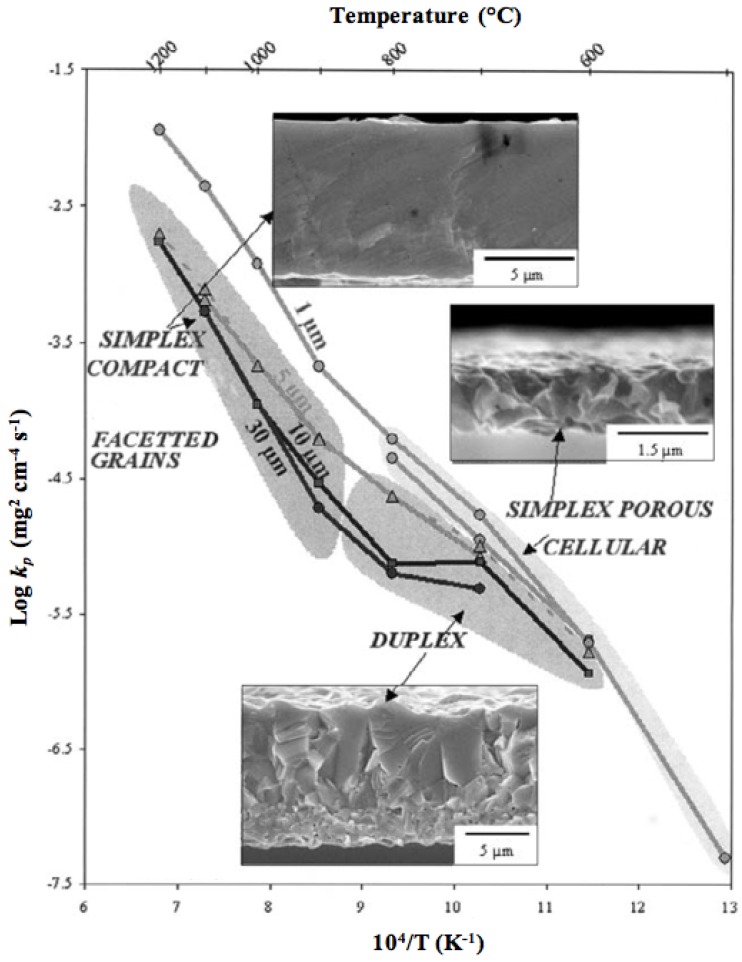
Arrhenius plot of parabolic rate constant *k_p_* as a function of oxidation temperature and scale thickness indicating NiO scale morphologies and microstructures [48,49].

For thicker oxide layers (>0.1 µm), oxidation is governed by ion diffusion through the oxide scale, which follows a parabolic behavior (Wagner theory), with *y*^2^ = *k_p_·t*, where *y* is the oxide layer thickness, *t* the time and *k_p_* the parabolic rate constant. The Ni^2+^ cation diffusion in its oxide is about 6 orders of magnitude faster than the oxygen anion diffusion at 1400 °C. This can cause internal voids during the growth of the oxide layer. For large surfaces, the inward diffusion of oxygen occurs through microcracking or microchannel formation. The microstructure of the oxide surface and cross-section varies depending on the oxide thickness and the oxidation temperature as shown by Peraldi *et al.* (see Figure 2) [48,49].

In the case of small metal particles, it was observed by focused ion beam cross-sectioning that internal porosity is formed due to different diffusion coefficients between Ni^2+^ and O^2−^ in the nickelous oxide like a pseudo Kirkendall effect (a pure Kirkendall effect is for a metal solid solution; in the case of NiO, Ni^2+^ and O^2−^ are on different crystallographic positions). Up to 1000 °C, outward diffusion of nickel cations is faster than the inward diffusion of oxygen anions, leaving NiO internal porosity [50]. For metal nanoparticles, the number of voids and the void’s growth depend on the relative rate of self-diffusion in the core material (*i.e.*, Ni or vacancy diffusion in Ni crystal) versus cation diffusion through the shell (*i.e.*, Ni^2+^ diffusion in NiO crystal) [51]. If self-diffusion is fast, a single void may form inside the particle and grow until conversion is completed (e.g., NiO). Alternatively, if self-diffusion is significantly slower than cation diffusion through the oxide shell, then several voids remain (e.g., CoO and Co_x_S_y_) [52]. For the Ni case, the particle size plays a role: smaller nanoparticles show single voids compared to larger nanoparticles presenting multiple voids. For the larger nanoparticles, the Ni self-diffusion is not fast enough to condense all the vacancies into a single void [53]. The key factor in microstructural modification during oxidation is the difference in diffusion coefficients defining the mass transport. 

By decreasing the oxygen partial pressure, the oxidation rate should decrease proportionally to *k_p_* = *C* × (*p*_O2_)^1/6^ (with *C* a constant). 

**Figure 3:**
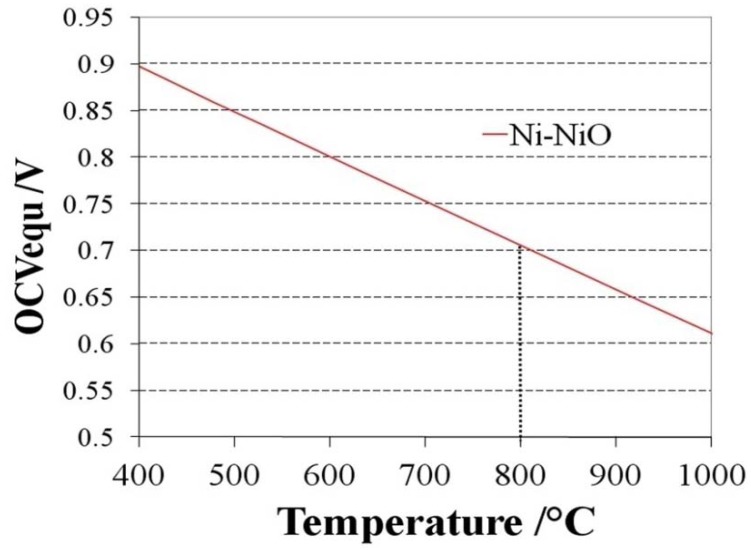
Open circuit voltage (OCV) or Nernst potential versus temperature for the Ni/NiO equilibrium [54,55].

The equilibrium partial pressure of oxygen can be calculated using the Gibbs free energy of the nickel oxidation reaction (Ni + ½ O_2_ ↔ NiO). From this a Nernst potential can be calculated against an electrode in air depending on the temperature:
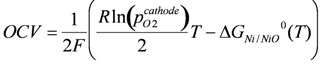
(4)
where *T* is the temperature in Kelvin, *R* the gas constant, *F* the Faraday constant and *p_O2_^cathode^* the partial pressure of oxygen at the cathode side. The value of the Nernst potential (or open circuit potential, OCV) versus temperature during Ni oxidation is given in Figure 3: at 800 °C, the OCV is between 0.68 and 0.71 V depending on the chosen database for the Gibbs free energy [54,55].

Solid state diffusion is activated by temperature as expressed by the Arrhenius Equation (3). Values of the activation energy are given in Table 3. It is observed that the rate at low temperature is higher than predicted by the exponential approach. At low temperature, the metal ions diffuse through the NiO grain boundaries and linear defects (dislocation and twins). Thus the oxidation rate will depend on the grain size in the oxide layer [47]:
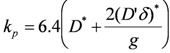
(5)
where *g* is the grain size, D^*^ the diffusion coefficient of Ni^2+^ in the NiO lattice, *D*' the diffusion coefficient of Ni^2+^ at the NiO grain boundaries and *δ* the thickness of the NiO grain boundaries (about 1 nm).

**Figure 4:**
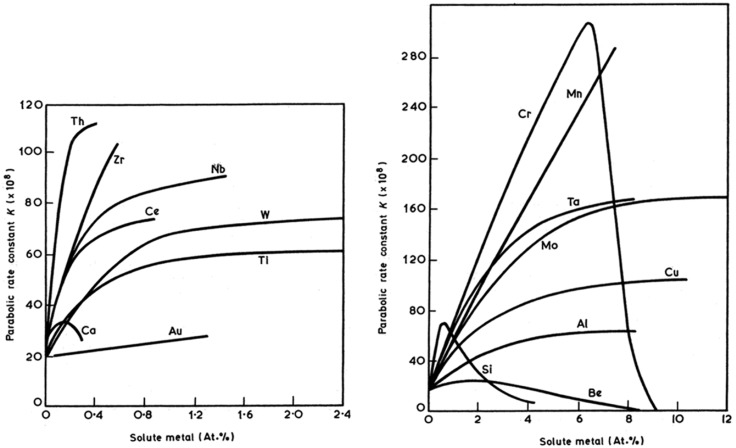
Effect of alloying on the rate constant for oxidation of nickel in air at 900 °C [46].

**Figure 5:**
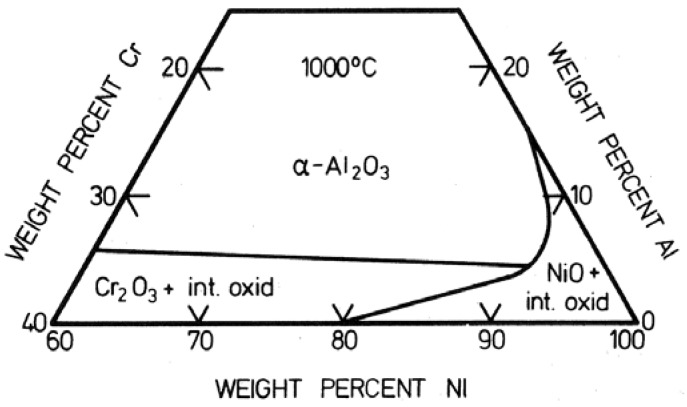
Oxide map for alloys in the Ni-Cr-Al system delineating the composition ranges for formation of different types of oxide scales [25].

Alloying the nickel increases the oxidation rate constant (see Figure 4). If the alloying element concentration is high enough to form a dense protective layer, the rate constant decreases (see Si and Cr in Figure 4). The simultaneous addition of two alloying elements can form a stable oxide layer at lower overall weight concentration than a single element (see Figure 5). 

**Table 3:** Oxidation kinetics of nickel and nickel cermet in air (with Ø for particle diameter).

Source Reference	Sample	Temperature range (°C)	E_a_ (kJ mol^−1^)	Kinetics
**Pure nickel**				
Suwanwatana *et al.* [56]	Ni particles, Ø = 79 nm	250–350	150	Deviation from parabolic
Suwanwatana *et al.* [56]	Ni particles, Ø = 0.7 µm	250–350	127	Deviation from parabolic
Suwanwatana *et al.* [56]	Ni particles, Ø = 3 µm	250–350	108	Deviation from parabolic
Karmhag *et al.* [57]	Ni particles, Ø = 15 nm	135–235	129	Deviation from parabolic
Karmhag *et al.* [58]	Ni particles, Ø = 5 µm	300–700	145	Deviation from parabolic
Karmhag *et al.* [59]	Ni particles, Ø = 158 µm	500–700	183	Deviation from parabolic
Haugsrud [60]	Polycrystalline Ni-mechanically polished	500–800	150	Deviation from parabolic
Karmhag *et al.* [59]	Ni particles, Ø = 158 µm	800–1200	116	Deviation from parabolic
Haugsrud [60]	Polycrystalline Ni-mechanically polished	1100–1300	200	Parabolic
Peraldi *et al.* [61]	Polycrystalline bulk Ni-mechanically polished	1000–1200	200	Parabolic
**Nickel–ceramic composite**				
Waldbillig *et al.* [41]	Tape-cast NiO-YSZ	500–850	87–92	Deviation from parabolic
Tikekar *et al.* [36]	Pressed NiO-YSZ rectangular bar	600–800	–	Parabolic
Stathis *et al.* [62]	Warm pressed NiO-YSZ	550–650		Logarithmic
Modena *et al.* [40]	Tape-cast NiO-YSZ	700–800	37–44	Logarithmic
Pihlatie *et al.* [38]	Tape-cast NiO-YSZ	500–1000		Linear–parabolic–logarithmic part
Roche *et al.* [41]	Tape-cast NiO-YSZ	600–1000	118	Deviation from parabolic
Czerwinski *et al.* [63]	Polycristalline Ni with CeO_2_-mechanically polished	600–800	88	–
Czerwinski *et al.* [63]	Polycristalline Ni with CeO_2_-chemically polished	600–800	100	–
Galinski *et al.* [64]	Thin sprayed NiO-40CGO	500–575	164	Parabolic–cubic
Galinski *et al.* [65]	Thin sprayed NiO-CGO	500–575	270	Mott–Cabrera equation for spherical geometries

### 2.3. Reduction of NiO-YSZ Cermet

The composite structure of the as-sintered NiO-YSZ anode changes the behavior during reduction and re-oxidation, compared to pure Ni/NiO.

Comparison of the reduction behavior of original NiO powder and NiO-YSZ anode during heating under reducing atmosphere in a thermogravimetric analyzer (TGA) shows a higher starting temperature and a slower rate for the composite structure [66]. By contrast, *in situ* transmission electron microscopy (TEM) shows a NiO-reduction starting at the NiO-YSZ interface. This contradictory result may come from the lower hydrogen pressure in the TEM and the different surface defects between TEM and bulk samples [67].

Tikekar *et al.* performed reduction on dense NiO-YSZ fabricated by compaction and measured the reduction rate by measuring the reduced layer thickness versus time. They found linear kinetics with an activation energy of 94 kJ/mol [36]. Waldbillig *et al.* performed reduction of tape-cast samples in a TGA and measured the activation energy (*E_a_*) by constant heating rate and constant temperature reduction and found a similar activation energy as for NiO powder, showing that the gas diffusion in NiO-YSZ dense samples is not limiting the kinetics (see Table 1) [41]. Pihlatie *et al.* observed also linear kinetics up to 80% NiO conversion with similar *E_a_* (between 500 and 750 °C) [38]. At high temperature (between 750 and 1000 °C), they reported a small decrease in the reduction rate by adding 3% water vapor to 9% H_2_ in N_2_ and attributed it to coarsening of the Ni phase under water vapor.

Grahl-Madsen *et al.* observed an increase in electrical conductivity by increasing the temperature of reduction [68]. The high conductivity could not be obtained by increasing the temperature after an initial reduction at lower temperature. This shows that the Ni phase is highly mobile during reduction of NiO.

Li *et al.* measured the performance of anode-supported cells against temperature of reduction (between 550 and 750 °C) and found for their case an optimal reduction temperature for 650 °C [69]. Jung *et al.* proposed to carry out the reduction via the ionic current (applying external potential to drive the O^2−^ from the NiO based electrode through the electrolyte) to enhance cell performance [70].

Pihlatie *et al.* observed a transient shrinkage (0.08%) of the composite sample during reduction at lower temperature (600 °C), which is due to the contraction of NiO to Ni [38,71]. At higher temperature the stress in the Ni phase is released by creep. The shrinkage of the Ni-YSZ composite depends on the temperature, with 0.04%, 0%, 0.01%, 0.05% and 0.3% of shrinkage after 15 h of reduction at 600, 750, 850, 1000 and 1100 °C, respectively [72]. It also depends on the as-sintered porosity of the sample: no shrinkage was noted up to 14% porosity, but 24% and 33% porosity led to 0.01% and 0.03% of shrinkage after 10 h of reduction at 850 °C, respectively [73]. The sample composition plays a big role: coarse NiO and YSZ powders could show up to 2.1% shrinkage after 25 h at 850 °C, while the addition of 20% of fine YSZ reduced the shrinkage to 0.5% for the same conditions [68].

Multiple RedOx cycles lead to a faster reduction rate after the first reoxidation [27,74,75]. This is due to the opening of the microstructure by breaking the thin electrolyte and the YSZ backbone and the change in the nickel oxide nano- and micro-structure. This will be described in more detail in the next section. Temperature programmed reduction showed that reoxidized NiO re-reduced at lower temperature than before the reoxidation, confirming the change in NiO nanostructure [76].

### 2.4. Oxidation of Ni-Ceramics Composite

This section reports observations during oxidation of the Ni-YSZ composite as described in the literature. The nomenclature introduced by Ettler *et al.* includes “external parameters”, like temperature, incident oxidant flow, duration of oxidation and gas flow rate, versus “internal parameters”, which are linked to the Ni-YSZ design, microstructure and composition [77]. This section will discuss the external parameters.

#### 2.4.1. Kinetics of Oxidation

In comparison to pure Ni (reduced in similar conditions), the composite starts to oxidize at lower temperature and with a faster rate [66].

An early study on *in situ* reduction of NiO-YSZ in a X-ray diffractometer showed that isothermal reoxidation at 600 °C is faster than the reduction at the same temperature [78].

The study of the oxidized layer thickness *vs.* time showed a parabolic behavior during re-oxidation of Ni-YSZ, indicating a diffusion-controlled process. As this process is not thermally activated, the conclusion is that the limiting rate is the diffusion of oxygen gas through the oxidized layer with an effective diffusion coefficient of 10^−7 ^cm^2^/s [36]. A TGA study observed a parabolic behavior at low temperature (400–650 °C) and a divergence from parabolic behavior between 700 and 850 °C, the activation energy being lower than the usual values observed for pure Ni (see Table 2) [41]. Other studies show logarithmic behavior of the oxidation of Ni-YSZ cermet activated by temperature at 550–650 °C [62] and 700–800 °C [40]. The difference in these results could come from the fact that the limiting process changes from solid state diffusion at lower temperature to gas phase diffusion at higher temperature, due to pore closing when Ni changes to NiO at the composite surface. The ideal-gas law gives a diffusion coefficient proportional to *T*^3/2^ [79] (high temperature cermet oxidation), compared to solid-state diffusion following the Arrhenius law (proportional to exp(−*E_a_*/*RT*), for low temperature cermet oxidation). Pihlatie *et al.* show a change in activation energy around 750 °C [38], whereas Roche *et al.* observe this transition around 800 °C in 20% O_2_. At lower p_O2_, the transition occurs at a lower temperature: with 1% O_2_, the gas diffusion is limiting the oxidation down to 600 °C [80]. The porosity of the support plays a role in the oxidation kinetics and makes the direct comparison of the different studies problematic, due to their different microstructures. 

Kinetics of oxidation of a Ni-Gd_0.4_Ce_0.6_O_2_ (40CGO) anode was studied by *in situ* X-ray diffraction between 500 and 575 °C, where a transition between parabolic to cubic behavior was observed. The time for full oxidation of nickel in the anode is 4 min at 650 °C and only 0.5 s at 850 °C [64] (the anode thickness was not given but it can be estimated to around 25 µm from a parallel study [81]). A following study fitted the oxidation kinetics between 500 and 575 °C of Ni-CGO composite with a Mott–Cabrera equation for spherical geometries. The higher activation energy compared to other studies (see Table 3) should be related to the compressive stress built up in the composite [65].

Multiple RedOx cycles showed a faster rate for the second reduction and second oxidation [27,74,75]. This is closely related to the faster reduction process: higher gas diffusion in the more open microstructure due to cracks in the YSZ electrolyte and skeleton, and finer Ni grains after the first cycle, as the oxidation is inversely related to the grain size (see Equation (5)). A temperature programmed oxidation study also revealed a finer Ni microstructure after a RedOx cycle [82].

#### 2.4.2. Homogeneous Versus Inhomogeneous Oxidation

The change in cermet oxidation kinetics with temperature can be linked to the transition from “homogeneous” to “inhomogeneous” oxidation. The first observation of homogeneous oxidation appears at low temperature (550–650 °C) under dry air where the full anode layer starts to oxidize homogeneously. By comparison, between 900 and 950 °C under Ar with 40% to 80% water vapor, oxidation starts at the surface and then moves inward with time. In inhomogeneous oxidation, a sharp border between oxidized and reduced side of the sample can be observed. This inhomogeneous oxidation leads to a warping or bending of the composite sample [62]. Further studies showed that oxidation under air between 700 and 800 °C also presented “inhomogeneity” and bending of the samples, compared to lower temperature oxidation (600 °C) [14,77,83]. At low temperature, oxidation kinetics is limited by the diffusion in solid state. At higher temperature, the limitation comes from gas diffusion through the re-oxidized layer, which has high tortuosity and low porosity. These observations can be compared to the results from Tikekar *et al.* [36], where they measured the thickness of the oxidized layer in air versus time, down to a temperature of 650 °C. Intrinsically, the sample oxidation has to be inhomogeneous in order to perform the measurement of oxidized layer thickness versus time. Only the gas limitation kinetics can be observed using this technique. Therefore Tikekar *et al.* did not observe thermally activated oxidation [36]. As mentioned before, the ideal gas law gives a diffusion coefficient proportional to *T*^3/2^ [79] compared to solid-state diffusion following the Arrhenius law (proportional to exp(−*E_a_*/*RT*)). This can be related to the kinetics of the reaction: inhomogeneous oxidation corresponds to O_2_ gas diffusion limited oxidation (high temperature and low *p*_O2_) and homogeneous oxidation is related to solid state diffusion limitation (low temperature and high *p*_O2_).

Some authors observed oxidation inhomogeneity at 650 °C [36] and others only from 750 °C [14]. This is probably related to the cermet microstructure: Lower porosity samples show inhomogeneous oxidation and bending at lower temperature. 

By lowering the partial pressure of oxygen of the oxidizing flow (from 50% to 20% and to 0.1% in He), the inhomogeneity of oxidation (and the sample bending) increased [83,84]. 

#### 2.4.3. Expansion during Reoxidation

The volume expansion of the anode due to nickel oxidation creates stresses in the different layers (compression in the anode and tension in the electrolyte). The stresses are proportional to the expansion: an essential measurement for the anode RedOx stability is dilatometry during re-oxidation. Theoretically, an expansion higher than 0.2% will fracture the thin electrolyte in case of an anode supported cell (see Section 2.4.6 for more details) [12,85]. Expansion was also measured at room temperature after re-oxidation [76,86] but the TEC variation between NiO-YSZ and Ni-YSZ composite should be taken into account [87]; the maximal strain can occur during oxidation and not after completion [88]. Dilatometry was performed *in situ* during oxidation at the initial stage of Ni-YSZ studies [62,89,90]. In 1998, Mori *et al.* reported an important expansion during TEC measurement of a 35 vol % Ni-YSZ sample in air [87]: At around 900 °C the expansion strongly increased, by about 1.2% to 1.5%. They observed cracks at the 8YSZ grain boundaries. 

Stathis *et al.* observed an increase in expansion with oxidation temperature in air, from 0.27% to 0.54% at 650 and 800 °C, respectively [62]. This is confirmed by other authors: Pihlatie *et al.* observed an expansion from 0.19% to 0.28% and to 0.93% at 600, 800 and 1000 °C, respectively [72]. Klemensø *et al*. saw even much higher expansion, from 0.99% to 4.95% at 700 and 1000 °C, respectively [26]. The difference in expansion for similar conditions could be related to a difference in microstructure, as shown by Fouquet *et al.* [66] and Waldbillig *et al.* [41]. This will be discussed in more detail in the next section. 

The effect of nickel sintering in reducing atmosphere on the expansion during a RedOx cycle was confirmed by Pihlatie *et al*., who found a doubling of the expansion for a sample reduced for 4.5 h at 1100 °C, compared to a sample reduced for 5.5 h at 800 °C [72]. This confirms the suggestion proposed by Cassidy *et al.* in 1996 [10] and presented in the small model (Figure 1) shown by Klemensø *et al.* about ten years later [26]. 

The water vapor plays an important role also during reoxidation as shown by the increase of expansion from 0.68% to 0.96% at 850 °C under dry air resp. air with 6% H_2_O, though at 600 °C no difference was observed. The effect of humidity at 850 °C is similar to the effect of increasing temperature up to 1000 °C [72]. This is an important result as at high fuel utilization, the water vapor on the anode side can reach high values depending on the fuel (up to 80%–90% under pure hydrogen). Then reoxidation will occur under high water vapor concentration. 

A small expansion at 850 °C was observed at an oxygen partial pressure of 5 × 10^−12^ atm, which is about 50 times the equilibrium partial pressure of oxygen for the Ni/NiO couple at this temperature [72]. 

Usually, subsequent RedOx cycles present an irreversible behavior as the contraction is smaller than the expansion; the second oxidation therefore reaches a higher maximal cumulative RedOx strain (CRS_max_) than during the first oxidation [72]. This behavior is observed by other authors [26,66,76]. At lower temperature the behavior can be reversible [76].

Sarantaridis *et al.* presented a nearly linear behavior between the oxidation strain and the degree of oxidation (DoO) with a small shrinkage of 0.05% at 5% DoO and a maximum strain of 0.55% reached at a DoO between 90% and 95%. The second oxidation presents the same behavior with a shift to a higher strain of about 0.1%. They also observed a difference in strain after the first oxidation at 800 °C when the sample was measured at room temperature (i) with interruption during oxidation; (ii) without interruption and (iii) for *in situ* dilatometry measurement (high temperature). Results were 0.55%, 0.65% and 0.80%, respectively [50]. 

Another study used crack widths in the thin electrolyte and the porosity increase in the anode for the expansion calculation after multiple RedOx cycles. From temperature variation, a “RedOx safe” temperature could be extrapolated downwards to 550 °C for this microstructure at which the thin electrolyte will not crack upon RedOx cycling. This was confirmed experimentally, also for real stacks experiencing fuel supply interruption. Repeated RedOx cycles at 800 °C showed a stabilization of the RedOx strain after multiple cycles [85].

Based on dilatometry measurements, Pihlatie *et al.* proposed a model based on continuum mechanics to fit all experimental expansion data versus temperature. The simulation shows that during reduction at low temperature (600 °C), the contraction of the sample is due to the limited creep in the nickel at these temperatures. During reoxidation at low temperature, the model shows that pseudo-plasticity or micro-cracking occurs in NiO, that at 850 °C the 3 mol % Y_2_O_3_-tetragonal zirconia polycrystal (3Y-TZP) backbone fractures and that at 1000 °C the 3Y-TZP creeps and undergoes micro-cracking. The main limitations of the model are that (1) the strength of 3Y-TZP is back-calculated and (2) the critical stress for pseudo-plasticity in NiO is directly dependent on the value used for NiO fracture toughness and critical flaws in NiO. These unknown parameters basically allow the fit of any RedOx strain [71].

#### 2.4.4. Bending and Stresses in Half-Cell Samples (Anode Support)

Stresses in the layers are present due to the difference in thermal expansion coefficient (TEC) between the anode and the electrolyte. The TECs for pure 8YSZ, NiO and Ni are 10.3, 14.1 and 16.9 × 10^−6^ K^−1^, respectively [87]. For a standard composition containing 58 wt % NiO, which corresponds to 56 vol % NiO and 43 vol % Ni, the corresponding TEC is 12.3 and 11.5 × 10^−6^ K^−1^, for the oxidized and reduced states, respectively [87]. This means that the electrolyte, which shrinks less during cooling, will be under compression and the anode under tension with a maximal value at the interface with the electrolyte [91]. The stress and bending calculations were done for CGO-YSZ, by Atkinson and Selçuk, and gave good results with a “stress free” state around 1200 °C [92]. 

Stress measurements were done on NiO-YSZ half-cells with a thin 8YSZ electrolyte using X-ray diffraction (XRD) at room temperature: The thin electrolyte stress is about −560 MPa and does not vary when the cell is flattened for stacking. The reduction of the half-cell for 10 h at 900 °C reduces the stress by about 10% [91]. These results are comparable to those reported by Sumi *et al.* on NiO-3YSZ anode-supports (300 µm) with 10ScSZ electrolyte (20 µm). At room temperature, the as-sintered electrolyte stress is −400 MPa, in the reduced state −250 MPa, and −170 MPa in the reoxidized state. The thin electrolyte showed cracks after reoxidation. The compressive stresses after reoxidation are due to the cooling from the RedOx temperature to room temperature. The anode is under tension of 50–100 MPa at the interface with the electrolyte. *In situ* measurements in a high energy (70 keV) X-ray synchrotron beam showed that the stresses are released at 1000 K [91]. Reasons for the electrolyte internal stress change between reduced and oxidized states are: (1) At high temperature, the porosity increase in the reduced anode lowers its Young modulus [93] and (2) at room temperature, the TEC changes between the oxidized and reduced Ni-YSZ composite [87]. Another study reported higher compressive stresses measured on NiO-YSZ anode-supports and YSZ electrolyte using a similar XRD technique and microscopic strain in 5 µm electrolyte grains (with an advanced method and synchrotron radiation [94]): The values were −690 MPa, −600 MPa and about 0 MPa, for room temperature as-sintered, reduced and re-oxidized samples, respectively. The electrolyte residual stress at 800 °C was measured as −60 MPa [95].

**Figure 6:**
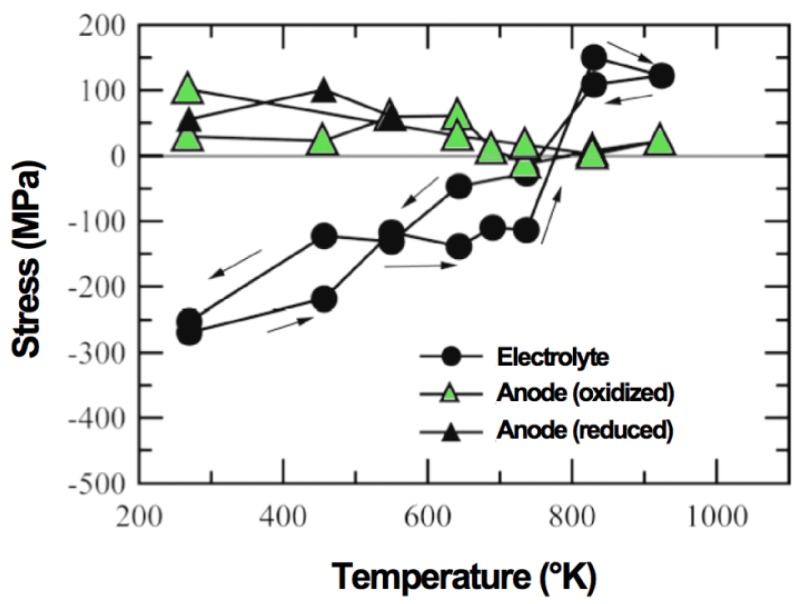
Changes in stresses in 10ScSZ (scandium-stabilized zirconia) electrolyte and anode during heating Ni-3YSZ under air [96].

Tanaka *et al.* presented the following work of Sumi *et al.* on similar samples. The *in situ* oxidation of Ni-3YSZ anode-supports during measurement showed a tensile stress in the electrolyte of 150 MPa at 800 K (see Figure 6). The Ni peaks disappear between 550 and 650 K when the nickel oxidizes. A difference of more than 100 K exists between the Ni peak disappearance and the tensile stress occurance [96]. The final stress in the electrolyte is similar to the one before the RedOx cycle; the thermal stresses are built up again, in contradiction to the stress release observed by Villanova *et al.* at room temperature [95]. 

**Figure 7:**
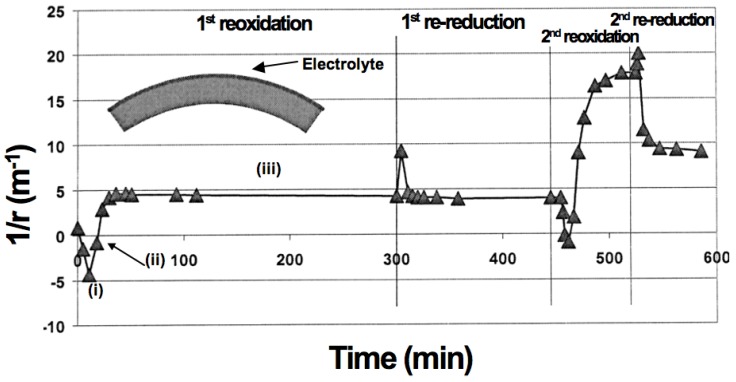
Curvature change during reoxidation and re-reduction cycles (0.27 mm half-cell, 800 °C) [97]. During re-oxidation, the half-cell shows (**i**) an initial curvature towards the electrolyte (on the concave side); then (**ii**) it reverts to “zero”-curvature and finally (**iii**) it stabilizes with a curvature towards the anode (on the concave side).

When oxidation occurs on one side, the half-cell bends due to chemo-mechanical expansion. This was observed *in situ* during oxidation at 800 °C of a half-cell composed of an anode-support, an active layer and a thin electrolyte of a total thickness of 0.27 mm [97]. As the oxidation starts at the anode, the cell bends towards the electrolyte (electrolyte on the concave side), then the curvature comes back to its initial value and bends further towards the anode (Figure 7). The authors explain the bending towards the electrolyte with an elastic deformation model but such a model cannot explain the bending towards the anode. Other authors propose plastic deformation of the anode during reoxidation to describe the anode being on the concave side after RedOx cycles [85,98]. 

**Figure 8:**
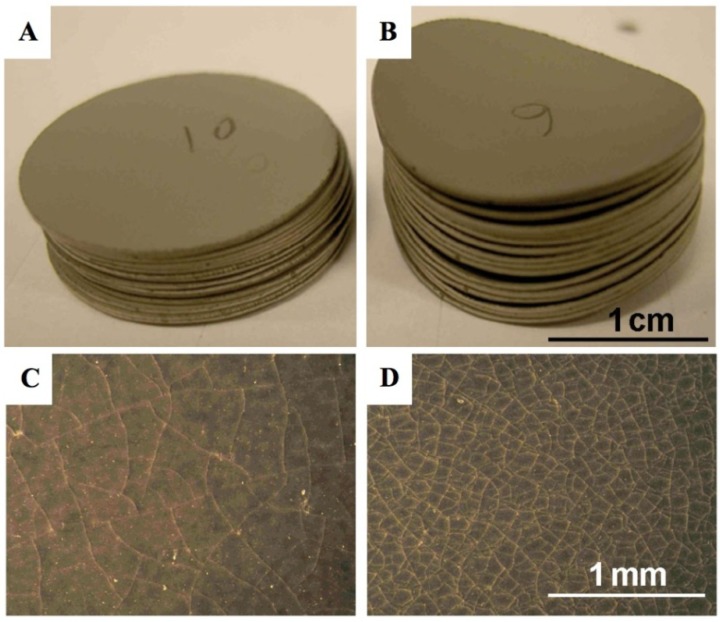
Picture of Ni-YSZ anode supported half-cell discs after 5 reduction-oxidation cycling (RedOx cycles) at 750 °C. (**A**) and (**C**): samples with 17.5% as-sintered porosity and (**B**) and (**D**): samples with 12% as-sintered porosity. The electrolyte is face-down for (**A**) and (**B**). A clear difference in curvature is observed between the two groups of samples comparing (**A**) and (**B**), (**B**) is bent towards the anode (anode face-up on the concave side). A clear difference in crack density is observed between the two groups of samples comparing (**C**) and (**D**) [98].

Other studies with NiO anode-supports with a 10CGO (10 mol % gadolinia-doped ceria) thin electrolyte showed bending with the anode on the concave side after reoxidation at 750 °C [99]. Ettler *et al.* showed that their NiO-YSZ (half-cell and full cell) bending towards the anode occurs at oxidation temperature higher than 700 °C, but that at lower temperature the bending is towards the electrolyte. They conclude that inhomogeneous oxidation bends the half-cell towards the anode (on the concave side, as shown in Figure 7) and homogeneous oxidation does the opposite [14]. Another *in situ* study revealed a curvature towards the electrolyte after re-oxidation at 800 °C [100]. By lowering the partial pressure of oxygen during reoxidation of a half-cell (at a constant temperature), the bending towards the anode is increased [83,84]. Half-cell samples with low porosity bent towards the anode (on the concave side) after 5 RedOx cycles at 750 °C, compared to higher porosity samples that stayed flat. The crack density in the thin electrolyte was higher in the case of the lower porosity samples as shown in Figure 8 [98].

Laurencin *et al*. studied the creep of the Ni-YSZ anode under reducing atmosphere. In the case of anode-supported cells (ASC), the compressive stresses decrease during the first 500 h of utilization from −220 to −120 MPa (based on creep measurement). This means that the RedOx tolerance of the thin electrolyte becomes lower with time as the compressive stresses in the electrolyte “work” against the RedOx anode strain. In the case of electrolyte-supported cells (ESC), the creep behavior will be beneficial by preventing the build-up of a high stress level after several RedOx cycles [101].

#### 2.4.5. Young’s Modulus and Strength Variation with Reoxidation

Young's modulus, strength and fracture toughness of NiO-YSZ and Ni-YSZ composites are well described by Atkinson and Selçuk [102] and Radoviç and Lara-Curzio [93]. The general trend is a decrease of the mechanical properties with increasing porosity (models are presented).

**Figure 9:**
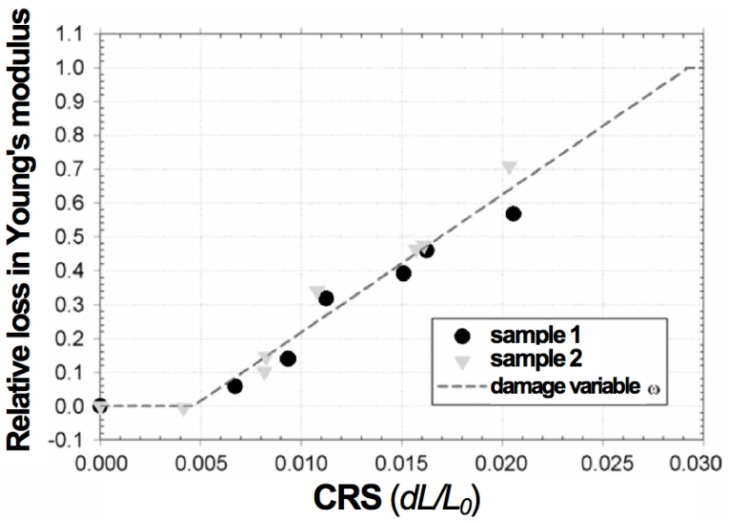
Mechanical degradation in terms of relative loss of elastic modulus of NiO-YSZ composite in its oxidized state during RedOx cycle as a function of the oxidation strain (CRS: cumulative redox strain). The measurement is reproducible (*i.e.*, samples 1 and 2) [103].

Pihlatie *et al.* used the impulse excitation technique (EIT) to study the evolution of Young’s modulus of NiO-YSZ with temperature in the as-sintered, reduced and reoxidized states [103]. They obtained a relation between the RedOx strain and the decrease in Young’s modulus. The damages caused by the RedOx cycles degrade the elastic properties. It starts linearly from 0.5% to 0.6% redox strain to macroscopic sample failure at 2.5%. An isotropic continuum damage model is given to fit the degradation: *E* = (1 − *w*) × *E*_0_ , with *w* the damage variable as a function of the oxidation strain (see Figure 9). 

Sarantaridis *et al*. showed a linear increase of the Young modulus (*E*) with the degree of oxidation (DoO), starting at 32 GPa and ending at 74 GPa [50]. The as-sintered *E* with 79 GPa is higher than for the reoxidized sample. The Young modulus is directly linked to the sample porosity [102], therefore the 5 GPa decrease can be related to a total porosity increase from 26.4% before to 27.6% after the RedOx cycle.

**Figure 10:**
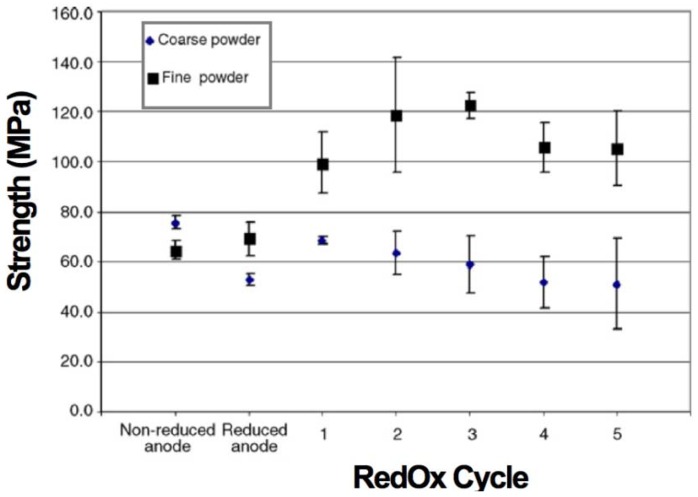
Strength of the C-shaped uniaxial compressed anode rings versus number of RedOx cycles. The samples were fabricated using two different powders, coarse green NiO and fine black nickel oxide [104].

Pusz *et al.* presented NiO-YSZ anode-supported tubular cells with an external diameter of 7.31 mm and a wall thickness of 1.7 mm. This study compared the strength evolution with RedOx cycles using ten samples each time of two compositions: (1) A fine structure containing black nickel oxide and (2) a coarse structure based on green NiO (*d*_v,50_ = 0.95 µm) with 40:40:20 vol % of NiO:8YSZ:Carbon pore former. Strength was measured at room temperature after 1 h RedOx cycling at 800 °C (see Figure 10). The strength of the fine microstructure sample doubled after 3 RedOx cycles. After the third RedOx cycle, the strength starts to decrease. The coarse microstructure showed a decrease in strength after reduction, a small increase for the first RedOx cycle and then a linear decrease with the RedOx cycle number [104]. 

Similar results were shown for planar anode supported cells after 10 RedOx cycles at 800 °C using 10 disc samples per measurement (25 mm of diameter). The mechanical strength of these supports increased slightly from 145 to 155 MPa after 10 RedOx cycles (the Weibull modulus also increased from 6 to 9) [105]. Another study on planar Ni-3YSZ half-cells showed no variation on strength after one RedOx cycle at 750 °C [98].

The authors were not very clear about the increase in strength during RedOx cycles. As maximal stress is located at the tube surface and the strength depends on the flaw distribution, this evolution can be linked to the surface change during RedOx cycles: densification of the surface lowers the flaw size at the surface. 

#### 2.4.6. RedOx Expansion Limits: Mathematical Approaches

Sarantaridis and Atkinson presented an analytical approach based on the release of stored elastic energy under plane strain conditions for modeling the maximal strain of the anode during RedOx cycles in case of anode-supported cells (ASC), electrolyte-supported cells (ESC) and inert substrate or metal-supported cells (MSC) [106]. 

The maximal strain of planar ASC before cracking the thin electrolyte can be deduced from Equation (6):

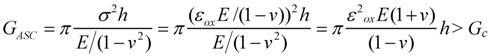
(6)
with *E* the Young modulus of the electrolyte, *v* the Poisson ratio, *h* the electrolyte thickness, *ε_ox_* the oxidation strain, *G_ASC_* and *G_c_* the stored and the critical energy release rate, respectively. 

Substituting the typical values given in [106], the product *ε^2^_ox _h_c_* = 7.4 × 10^−12^ m is a constant, with *h_c_* the critical thickness when *G_ASC_* = *G_c_*. The interesting point is that a decrease in electrolyte thickness increases the RedOx stability (more RedOx strain possible). Thus, for a given oxidation strain of 1%, the critical thickness is as small as 0.074 µm. An electrolyte thickness of about 2 µm gives a RedOx strain limit of 0.2%. In case of elastic relaxation, the crack spacing, *l*, is given by 8*h/*ln(*h/h_c_*), which means no extensive damage will occur until *h_c_ <* 2*h*. Hence an electrolyte of 4 µm thickness could be tolerated.

For the electrolyte-supported configuration, the failure mode will be delamination of the anode. Based on 8YSZ with a certain porosity, delamination occurs if the anode exceeds 2.6 µm; for a 10 µm anode, an oxidation strain of 0.5% can be tolerated. Buckling of the anode requires an initial delamination of 170 µm in size. Decreasing the thickness increases the strain linearly; hence a thin anode layer is more RedOx stable. 

**Figure 11:**
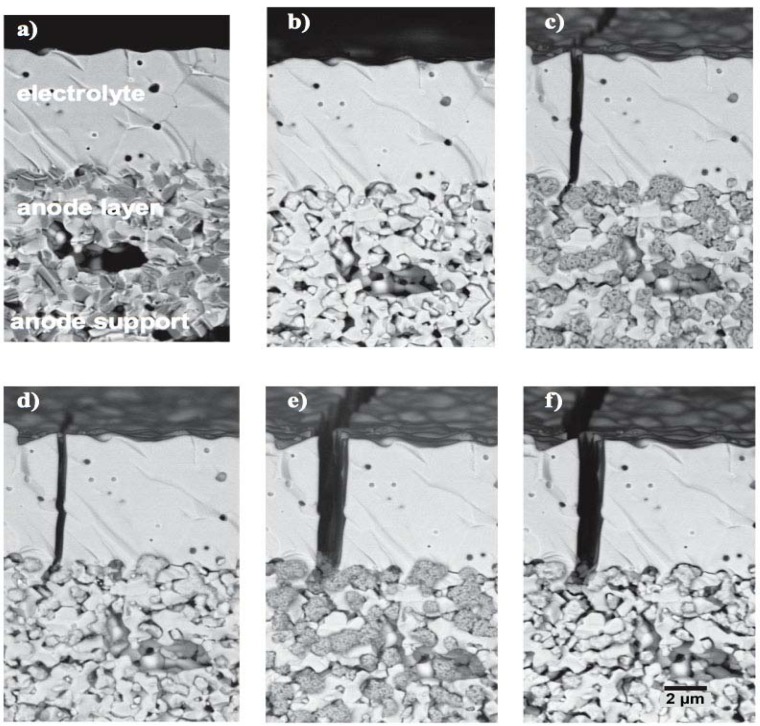
Thin electrolyte crack formation during two RedOx cycles in the anode supported cell design. (**a**) co-firing; (**b**) reduced (**c**) re-oxidized; (**d**) second reduction; (**e**) second oxidation and (f) third reduction with an additional 100 h under reducing atmosphere [14,107,108].

**Figure 12:**
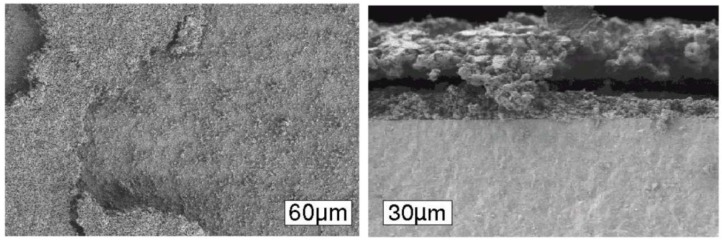
Delamination of anode and anode current collection layer in case of 8YSZ electrolyte supported cells after five RedOx cycles at 950 °C and 40 min. Right: Only Ni-8YSZ active anode; left: active anode plus Ni-8YSZ current collecting layer [16].

Cracks in the thin electrolyte for ASC configuration (Figure 11) and delamination of the anode for ESC (Figure 12) are shown by other authors confirming the degradation mechanisms proposed by Sarantaridis and Atkinson. 

For a metal support, in case of edge initiation delamination with a thickness of 10 µm each for the cathode, the electrolyte and the anode layers, a limit of 1% strain can be obtained. 

In summary, the maximum RedOx strain before degradation is: 0.2% for ASC, 0.5% for ESC and 1% for MSC. ASC is the most sensitive geometry in terms of RedOx stability, not only because it is breaking the gas tight electrolyte, but also due to the layer configuration.

Klemensø [27] and Klemensø and Sørensen [109] proposed an approach including anode support (AS), active functional layer (AFL) and electrolyte for the ASC case. Usually, the AFL has a finer microstructure to enhance electrochemical performance whereas the AS serves proper mechanical stability, sufficient electrical conductivity and gas transport properties. Lowering the temperature and decreasing the anode support thickness will increase the RedOx stability. For AS thickness of 300 µm, AFL and electrolyte thickness of 10 µm, the maximal strains before electrolyte cracking are:

1. at 650 °C, 0.2% for AS and 0.7% for AFL;
2. at 800 °C, 0.2% for AS and 0.25% for AFL.

**Figure 13:**
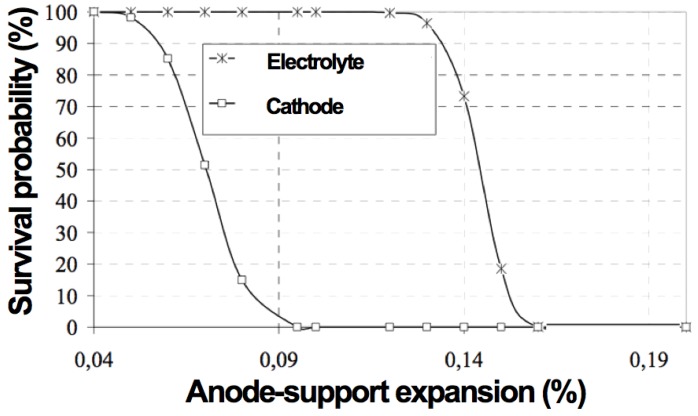
Survival probability of LaSrMn-oxide (LSM) cathode (60 µm) and YSZ electrolyte (10 µm) against the strain of the Ni-YSZ anode support (1 mm) [110].

Based on finite element modeling (FEM) calculations and failure probabilities of a Ni-YSZ anode-supported cell with 1 mm thick support, 10 µm thick 8YSZ electrolyte and 60 µm thick LSM cathode with a cell diameter of 116 mm, it has been shown that the cathode will crack when the support expands by more than 0.05%–0.09% and when the electrolyte expands by more than 0.12%–0.15% (see Figure 13) [110]. 

Some singularities are considered in the modeling and give hints for the fabrication, e.g., that the cathode/electrolyte contact angle at the cathode side in an ASC should be higher than 90° to increase stability [111]. The ESC configuration with a 10 µm thick anode will delaminate after 0.3% to 0.35% expansion. Experimental results based on the ASC from the Forschungszentrum Jülich (FZJ) showed that the electrolyte cracked after a degree of oxidation between 56% and 70.7% at 800 °C (similar to the value obtained by Sarantaridis *et al.* [112]), which corresponds to an expansion between 0.26% and 0.34%. Modeling underestimated the maximal value of expansion, which could be due to the inhomogeneity of oxidation at 800 °C in that only the anode side opposite to the electrolyte was oxidized. Cracks in the electrolyte were quantified by SEM and permeability of the electrolyte and expansion were measured using micro-Vickers marks distance before and after expansion [113]. Based on Weibull statistics and FEM, it can be seen that sample size influences the maximal anode strain before ACS thin electrolyte cracking, from 0.18% for small samples (0.1 cm^2^) to 0.12% for total stack surface (2000 cm^2^) [85].

Sarantaridis *et al.* compared the oxidation in air with oxidation by ionic current at 800 °C. They proposed a model that takes into account the non-uniformity of the electrochemical reoxidation on the failure probability of the electrolyte. The critical degree of ionic current reoxidation occurs at 3% (compared to 49% to 75% by oxidation in air), it creates a compressive stress in the central reoxidized anode located under the cathode and a radial tensile stress in the non-reoxidized anode [112]. 

#### 2.4.7. Electrical Conductivity versus RedOx Cycles

Robert *et al.* tested 800 µm thick anode-supports produced by slip casting with a porosity gradient created by sedimentation during the production process. The conductivity was measured on 120 mm diameter cells at 900 °C: it decreased from 2400 to 1300 S/cm after 7 RedOx cycles [90].

A doubling in electrical conductivity was observed after a RedOx cycle at 850 °C of a Ni-YSZ composite based on coarse YSZ (from 500 to 1000 S/cm). After conductivity decrease due to nickel coarsening, the experiment was repeated on the same sample and the conductivity rose back to the highest level. Grahl-Madsen *et al.* reported that conductivity degrades faster after RedOx cycling [68]. 

**Figure 14:**
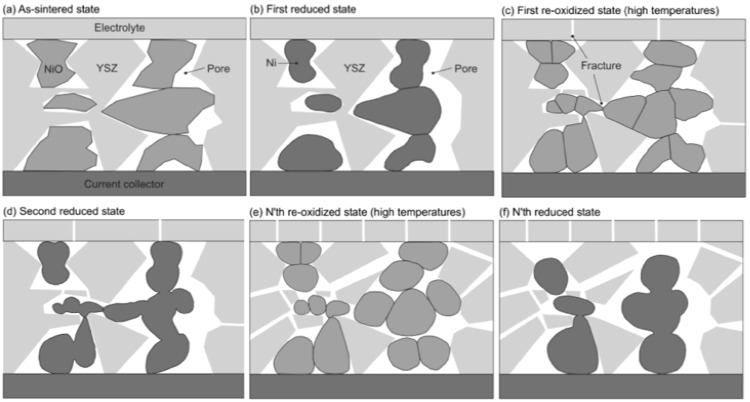
Model proposed by Klemensø *et al.* including the increase of Ni contact after a RedOx cycle due to breaking of the zirconia skeleton. Further RedOx cycles will create too much porosity to maintain sufficient conductivity [114].

Ni-YSZ samples produced by tape-casting showed a conductivity decrease after the initial reduction [114]. After the first RedOx cycle at 1000 °C, the conductivity increases to a value higher than the original value. After multiple RedOx cycles, the conductivity decreased and the degradation was faster than after the initial reduction. After removing Ni from the cermet with acid leaching, the ionic conductivity of the YSZ cermet was measured and showed a decrease due to cracks produced in the YSZ backbone by the RedOx cycle. A new proposed model included the increase in Ni contact after a RedOx cycle due to breaking of the zirconia skeleton. Further RedOx cycles will create too much porosity to maintain sufficient conductivity (see Figure 14). 

The conductivity of Ni-YSZ was measured at different temperatures and atmospheres (dry, wet or diluted hydrogen) [21]. At 600 °C under wet hydrogen, the conductivity starts at 1200 S/cm and is constant over 150 h. RedOx cycles increase the conductivity to 2300 S/cm. At 850 °C under dry 40% H_2_ (diluted in He), the conductivity degraded by about 35% over 200 h. After a RedOx cycle, initial conductivity was restored at first. After the RedOx cycle, the conductivity degradation with time is lower over the same time period. An interesting point is that the dilution of dry hydrogen has an influence on conductivity losses, with a faster degradation in the case of He-dilution compared to Ar-dilution.

**Figure 15:**
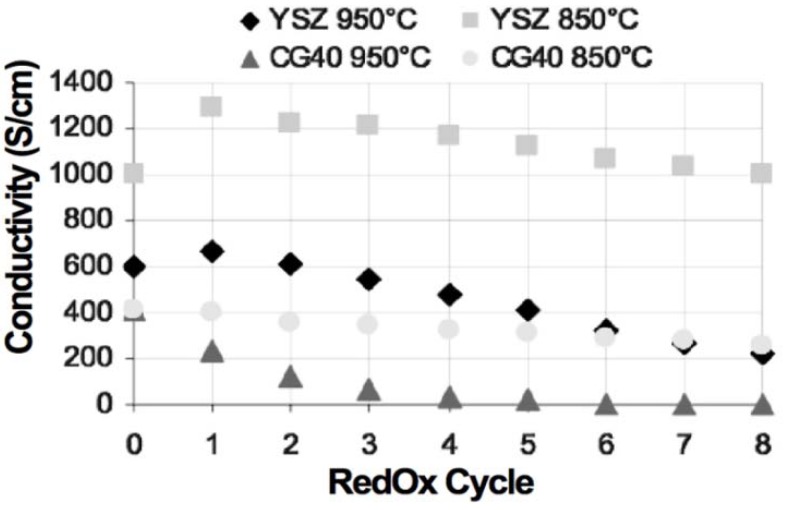
Comparison between Ni-YSZ and Ni-CGO composite electrical conductivity under RedOx treatments [81].

A comparison of electrical conductivity for Ni-8YSZ and Ni-40CGO composites (on electrolyte supports) under RedOx treatments was performed by Iwanschitz *et al.* The conductivity was measured during 8 RedOx cycles at 850 and 950 °C: at higher temperature, the degradation was fast after 4 RedOx cycles and the Ni-CGO sample was not conductive anymore. In the case of Ni-YSZ, an increase in conductivity was observed during the first cycles (see Figure 15). The conductivity is always higher for Ni-YSZ than for Ni-CGO composites. The degradation after RedOx cycling is related to microstructure coarsening [81].

Liu *et al.* studied the conductivity of a NiO-YSZ anode of 800 µm thickness covered with a 10 µm thin YSZ electrolyte by electrochemical impedance spectroscopy (EIS) during reoxidation and re-reduction. EIS spectra were taken between 9 and 1000 kHz each minute during oxidation in air at 500, 600, 700 and 800 °C. The high frequency impedance spectra give the ohmic resistivity of the cell. The evolution in ohmic resistance during oxidation occurs in three phases: (1) low constant resistance; (2) a strong increase to a maximum value and (3) finally a decrease to reach an intermediate plateau. These stages correspond to the oxidation of the Ni particles until cutting the Ni conduction path followed by the creation of a new conduction path through NiO after volume increase (spongy-like porous NiO after reoxidation). The maximum ohmic resistance was reached after 3, 19 and 73 min at 800, 700 and 600 °C, respectively. No change was observed at 500 °C over 450 min due to slower kinetics. During reduction, the conductivity increases faster, meaning that the Ni network forms much faster [115].

#### 2.4.8. Temperature Variation during Oxidation

Pomfret *et al.* observed a 20 K increase of temperature during anode-support reoxidation (at around 725 °C under air) using near infra-red imaging [116].

With basic thermodynamic data, a temperature increase of 1678 K is calculated from the adiabatic reaction of an anode-support (final composition of 55 wt % NiO and 45 wt % YSZ) from Equation (7) [85]:

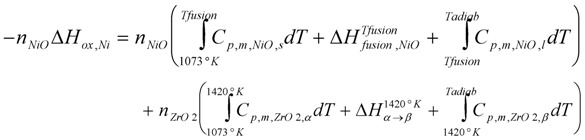
(7)
where *n_NiO_* and *n_ZrO2_* are the number of moles of NiO and zirconia, respectively, *C_p,m_* is the molar heat capacity (at a constant pressure), *∆H_ox,Ni_* is the enthalpy of nickel oxidation, *∆H_fusion,NiO_* is the fusion enthalpy of nickel oxide (the melting point of NiO is *T_fusion _*= 1990 K), *∆H_α→β_* is the enthalpy for zirconia phase change (from *α* to *β* phase) and *T_adiab_* is the calculated final temperature for the adiabatic reaction (*T_adiab_* = 2478 °C). *C_p,m _*is calculated from equation: *C_p,m_* = *a* + *bT*; the heat capacities for pure *α* and *β*-zirconia and solid and liquid NiO were used for the calculation [29]. Thermodynamic constants are given in Table 4. For the local anode temperature, the heat exchange with other parts and gases surrounding the anode should also be taken into account. This thermal effect can influence *in situ* expansion measurements (and even the furnace temperature, see Figure 16 in [117]), but it is nearly never taken into account in the different studies. After cracking the thin electrolyte, the combustion of the fuel at these locations creates hot spots with high water vapor that can induce accelerated nickel coarsening or cathode decomposition [85]. 

**Figure 16:**
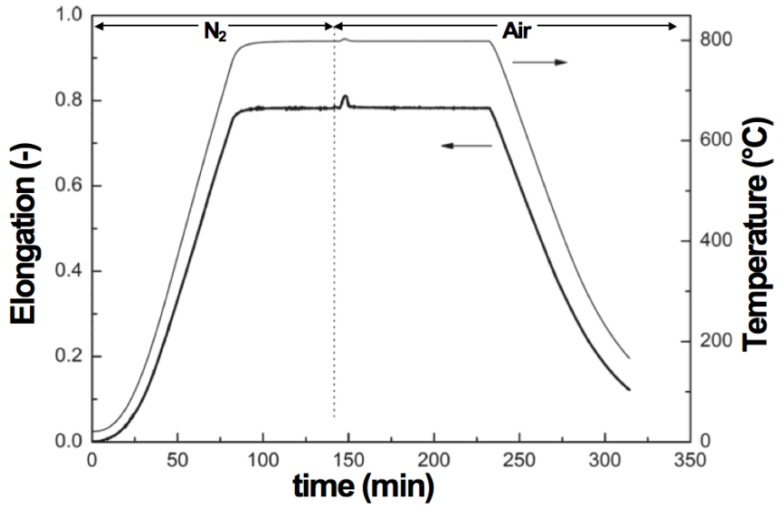
Dilatometry measurements of oxidation for a YSZ composite bar infiltrated with 16 wt % Ni [117].

**Table 4:** Thermodynamic constants for NiO and ZrO_2_. *C_p,m_* is the molar heat capacity (at constant pressure), ∆*H_ox_* is the enthalpy of nickel oxidation (Ni +1/2O_2_ → NiO at 800 °C), ∆*H_fusion_* the fusion enthalpy of nickel oxide (melting point of NiO is *T_f_* = 1990 °C) and *∆H_α→β_* the enthalpy for zirconia phase change (from *α* to *β* phase). *C_p,m_* is calculated from equation *C_p,m_* = *a + bT* [29].

	*a* (J mol^−1^ K^−1^)	*b* (J mol^−1^ K^−2^)	∆ *H_ox,Ni _* (kJ mol^−1^)	∆ *H_fusion _* (kJ mol^−1^)	*∆H_α→β _*(kJ mol^−1^)
NiO solid	46.81	8.46 × 10^−3^	239.8 [29]	50.66 [34]	–
NiO liquid	59.87	–	–	–	–
*α*-ZrO_2_	57.80	16.67 × 10^−3^	–	–	4.75 [34]
*β*-ZrO_2_	78.63	–	–	–	–

#### 2.4.9. Reoxidation by Ionic Current

The ionic current coming from the cathode side can oxidize the Ni if no fuel is available at the anode side, as in the following equation:


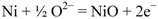
(8)

The charge (in C) is directly calculated by the multiplication of the current density (A/cm^2^) by the active surface (cm^2^) and the time (s). 

Hatae *et al.* observed spongy-like structures of the Ni-NiO phase closer to the anode/electrolyte interface for a sample oxidized under N_2_ at 800 °C (YSZ electrolyte and 8YSZ-NiO active anode and support). The current conditions were 7.5 mA/cm^2^ for 30 min, giving 54 C. X-ray stress measurements in the electrolyte showed a lower compressive stress under the cathode (−298 MPa) compared to the side of the cell (−324 MPa) and to a non-reoxidized cell tested in similar conditions (−339 MPa) [118]. 

Other studies from Hatae *et al.* reported contradictory results: one showed an activation of the electrochemical performance after oxidizing the anode with 15 C at 800 °C under nitrogen at current densities of 25 and 259 mA/cm^2 ^ [119]; another study presented degradation of the cell after a charge transfer of 15 C at a current density of 12.5 mA/cm^2^ [120]. In both cases, the open circuit voltage (OCV) was constant. In a recent study on anode-supported cells, Hatae *et al.* showed an increase of electrochemical performance of about 36% after reoxidation via ionic current (250 mA cm^−2^ and 15 C = oxidation of 0.6% of Ni anode and anode support). In the same time OCV increased by about 2%. After 17 such RedOx cycles, the performance slightly decreased due to delamination between the anode and the electrolyte and cracks in the YSZ anode network. Two longer oxidation periods at the same current density (equal to 31% oxidation of the Ni) showed a decrease in OCV (−2%) but with an increase in performance at 0.25 A cm^−2^ of +26% [121].

Sarantaridis *et al.* compared the oxidation in air with the oxidation by ionic current at 800 °C [112]. Due to the non-uniformity of the electrochemical oxidation, the critical degree of such oxidation occurs at 3%, compared to 49%–75% by oxidation in air. 

Increase of the ohmic and polarization resistances was observed after electrochemical oxidation of nickel from a cell from the Forschungszentrum Jülich [122]. As the peak frequency in the electrochemical impedance response and the OCV remained constant, the authors proposed a delamination-degradation mechanism occurring at the interface between anode support and active layer.

Takagi *et al.* studied the influence of humidity in nitrogen during oxidation by ionic current. They analyzed the microstructure of a Ni-YSZ anode on a 500 µm electrolyte (YSZ) by 3D reconstruction with dual beam SEM-FIB and measured the electrochemical performance after 2 electrochemical RedOx cycles under dry N_2_ and 20% humidified N_2_. The humidity during oxidation makes the particles more spherical, which lowers their connectivity and decreases the electrochemical performance. The oxidation under dry conditions makes the particle size increase without change in the shape. Degradation is thus much lower in case of dry reoxidation by ionic current [123].

#### 2.4.10. Micro and Nano-Structural Changes upon Redox Cycling

Macrostructural changes and physical property variations gave already some understanding on RedOx cycle effects on Ni-YSZ composite microstructures. 

The most used technique to observe post-microstructures is scanning electron microscopy (SEM). First observations showed coarsening of the NiO particles and microcracks in the YSZ skeleton [66]. Zhang *et al.* observed a sponge-like aggregate of NiO crystallites. The re-reduction of this microstructure led to coarse Ni particles, suggesting a re-dispersion inducing some transport of nickel and nickel oxide during RedOx cycling [76]. In parallel, Waldbillig *et al.* observed smaller pores in this sponge-like reoxidized NiO microstructure. In the same study, *ex situ* oxidation of a transmission electron microscope (TEM) lamella at 700 °C during 15 min showed nanometric polycrystalline NiO, even if the original nickel grain was a porous micrometric crystal [124]. An *in situ* environmental SEM study showed live re-oxidation of nickel-YSZ composite under low pressure of 5–10 mbar of air. Isothermal oxidation at 850 °C showed a rapid oxidation with a separation of original nickel grains in 2–4 smaller particles that grew in the voids and out of the polished plane. In case of a temperature ramp oxidation, the oxidation starts at around 300 °C and progresses slowly until 450–500 °C, at which point the rate increases. This procedure presents the formation of a protective nickel oxide surface layer around the original nickel particle. The microstructure will depend on the oxidation condition of the composite; even the partial pressure of oxygen, which is much lower in the case of *in situ* SEM observation, can change the microstructure evolution [17]. 

**Figure 17:**
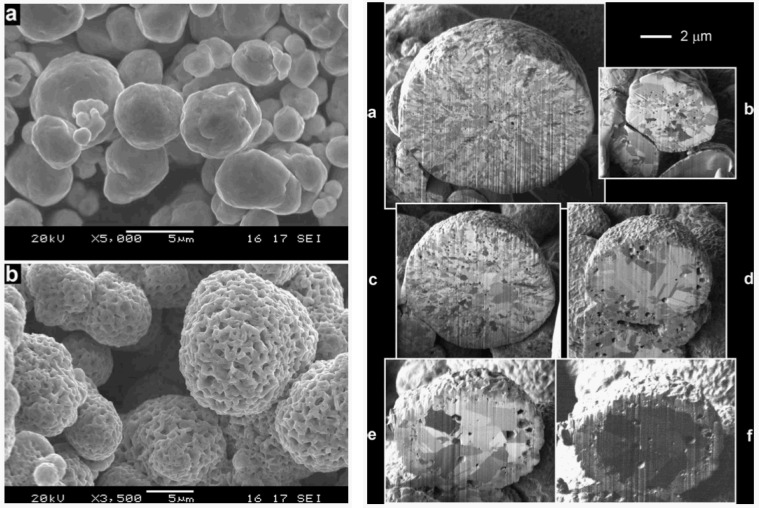
Left: SEM of (**a**) as received Ni and (**b**) fully oxidized Ni (NiO) particles. The secondary electron images were recorded using a beam energy of 20 keV. Right: FIB cross-sectional secondary electron images of Ni particles oxidized at 800 °C for (**a**) 30 s; (**b**) 60 s; (**c**) 90 s; (**d**) 180 s; and (**e**) 300 s. Image (**f**) is the same particle as in (**e**) but obtained using the secondary ion signal [50].

To understand the increase of the closed porosity by a factor of 3 before and after a RedOx cycle at 800 °C of a NiO-YSZ composite, Sarantaridis *et al.* used dual beam SEM-focused ion beam (FIB) to study the microstructure evolution of pure nickel particles of 5µm in diameter after oxidation at 800 °C. The surface of the sample using secondary electrons from the electron beam after oxidation is more textured and shows the sponge-like structure (see Figure 17 left). This effect is less pronounced when using the secondary electrons from the ion beam. To study the internal porosity, FIB was used to cut the particles after various oxidation times at 800 °C (see Figure 17 right). The evolution shows an increase of subsurface porosity during oxidation due to the outward diffusion of Ni^2+^ [50]. 

**Figure 18:**
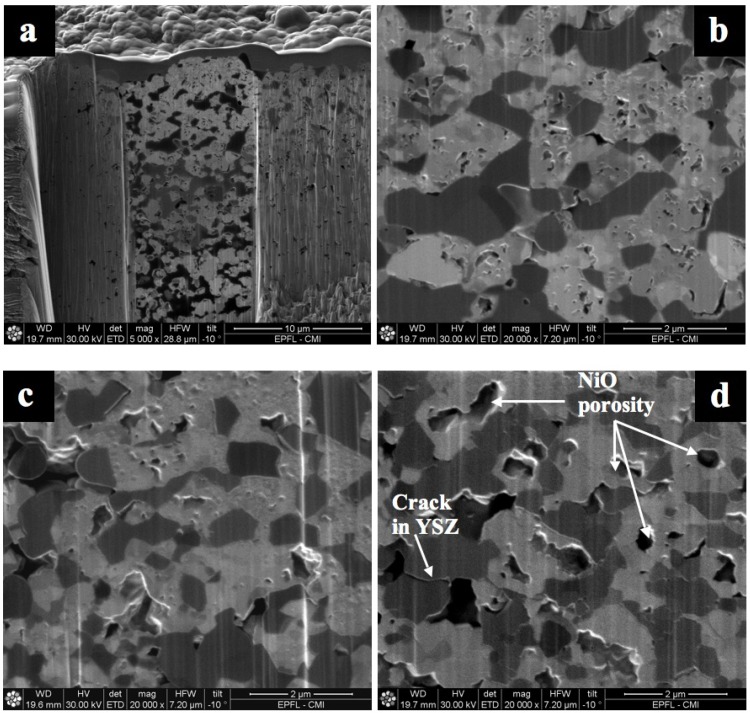
Secondary electron image from FIB cross-section from half-cells after one RedOx cycle (**a**) at 550 °C (lower magnification); (**b**) at 550 °C (higher magnification); (**c**) at 800 °C and (**d**) at 1000 °C. NiO contains small pores after a RedOx cycle at 550 and 800 °C but a single big pore after a RedOx cycle at 1000 °C. Dark grey is YSZ and light grey is NiO. The vertical lines come from the FIB milling process (“curtain effect”) [85].

Similar observations were done on Ni-YSZ composite reoxidized at 550, 800 and 1000 °C under air. Cross sections of the sample with SEM/FIB showed bigger NiO closed porosity at elevated temperature and small well dispersed NiO porosity at low temperature (see Figure 18) [85]. This change in NiO closed porosity can be related to the outward Ni diffusion process during oxidation. At relatively low temperature (*i.e.*, 550 °C), the Ni transport occurs via grain boundary of the NiO outer layer, and at elevated temperature (*i.e.*, 1000 °C) the Ni transport occurs through the NiO crystal lattice [47]. 

**Figure 19:**
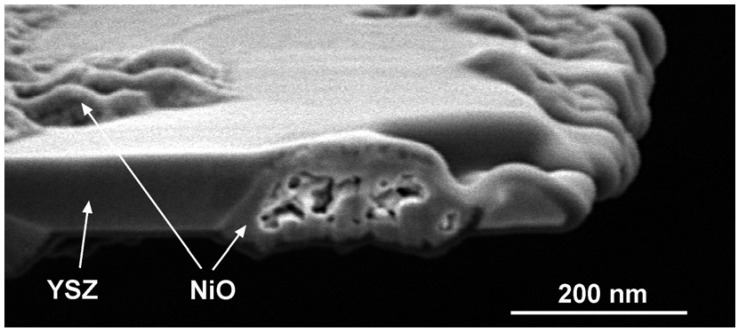
Cross-section of a transmission electron microscope (TEM) lamella after an *in situ* RedOx cycle, showing the hilly surface and closed porosity of the nickel oxide after reoxidation [129].

TEM observations showed porous NiO after an *in-situ* RedOx cycle. After the RedOx cycle, NiO grains grow out of the TEM-lamella plane and inside preexisting pores [125]. Cross-section observation of the tested TEM lamella shows closed porosity inside the NiO (Figure 19). These observations can explain an irreversible strain after a RedOx cycle due to the re-oxidation process that increases the nickel oxide closed porosity. Understanding better the nickel oxidation process shows that the nickel coarsening during anode utilization is not the only cause of Ni-YSZ anode instability.

**Figure 20:**
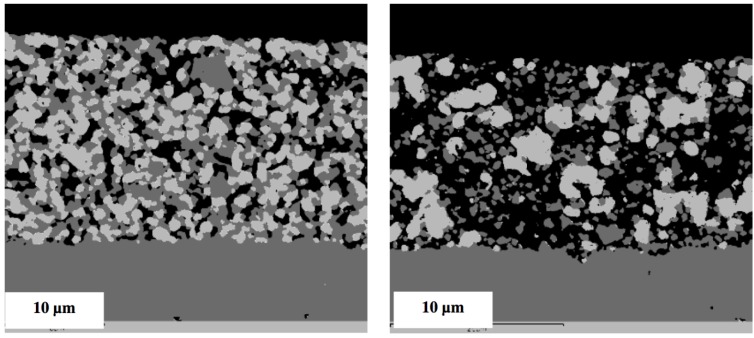
Fine Ni-8YSZ anode before (left) and after (right) eight RedOx cycles at 950 °C (SEM, backscattered electron detector, 10 kV) [126].

Multiple RedOx cycles at elevated temperatures destroy the Ni-YSZ microstructure of an electrolyte-supported cell. A strong increase in porosity and in Ni particle size was observed after the process in Figure 20 [126].

After an initial RedOx cycle, temperature programmed reduction (TPR) showed a lower temperature of reduction and a faster reduction rate [76]. X-ray diffraction (XRD) revealed a broadening of the NiO peaks [127,128]. These two observations confirm the decrease of particles and crystallites size during the Ni reoxidation process in a Ni-YSZ composite.

3-D reconstructions using FIB-SEM microscopy of oxidized Ni-CGO cermet at 510 and 575 °C showed nucleation of temperature-dependent pseudo-Kirkendall voids. Larger pores were observed at the highest oxidation temperature [65]. 

Microstructural evolution of Ni-YSZ composite was observed by X-ray computed tomography. Limited microstructural change was seen after 10 min oxidation steps at 500 °C but a porous NiO layer of about 700 nm was reported after 10 min at 700 °C [130].

#### 2.4.11. Electrochemical Performance and Electrochemical Impedance Spectroscopy

The electrochemical performance after a RedOx cycle can vary a lot. In case of ASC, a decrease in OCV can occur, indicating the thin electrolyte to crack [10,90,113]. 

The performance can increase due to, first, a better electrical contact between cell and current collecting layer [131] and, second, an activation [73,132] or re-activation after degradation [133] of the active anode. 

Pihlatie *et al.* observed a decrease in *R_p_* after a RedOx cycle at 650 °C and a small decrease in *R_s_* after a RedOx cycle at 850 °C, of symmetrical Ni-ScSZ anodes on a ScSZ electrolyte-supported cell. Microstructural observation revealed a finer microstructure after the 650 °C RedOx cycle and cracks in the electrolyte after the 850 °C RedOx cycle [73].

**Figure 21:**
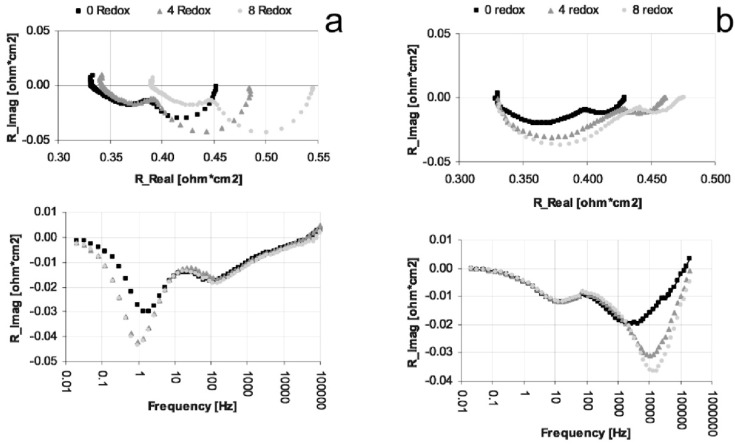
Impedance spectra at OCV during RedOx cycling at 950 °C of (**a**) Ni-40CGO (Ce_0.6_Gd_0.4_O_2−d_) and (**b**) Ni-8YSZ anodes with a 8YSZ electrolyte support. Top: Nyquist plot; bottom: complex impedance plot [81,126].

In many cases, the performances decrease due to an increase in polarization resistance (*R_p_*) [16,66,81,126,127,131] and in some cases an increase in ohmic resistance (especially for Ni-CGO, see Figure 21) [81,126]. 

Iwanschitz *et al*. showed the evolution of the imaginary part of impedance versus frequencies after RedOx cycles for Ni-8YSZ and Ni-40CGO [81,126] (see Figure 21). After a cycle at 950 °C, the Ni-CGO anode showed an increase of the peak at 1 Hz (corresponding to the conversion and diffusion impedances [134,135]) as well as of the ohmic resistance *R_s_*. This increase in peak height means a change in the gas transport process, while the *R_s_* evolution is linked to the electronic conductivity decrease in the Ni-40CGO layer (see Figure 15). The Ni-YSZ anode showed an increase of the high frequency peak (corresponding to the charge transfer impedance [136,137]) from 1 kHz to 10 kHz. The variation in frequency after the RedOx cycles means that the capacity layer between Ni and YSZ is changed. The change in peak height showed a degradation of the anode due to a decrease in active sites. 

A correlation study between the electrochemical characteristics and the microstructural evolution was done using 3D microstructure reconstruction with a FIB/SEM microscope. RedOx cycles of a Ni-YSZ thin anode on an electrolyte-supported cell at 1000 °C showed electrochemical performance degradation: Anode polarization losses increased from about 0.06 initially to about 0.09 Ω cm^2^ after the fourth RedOx cycle. This was correlated to a decrease in triple phase boundary (TPB) length of the anode from initial 2.49 to 2.11 µm^−2^ after 4 RedOx cycles [138,139].

Laurencin *et al.* studied Kerafol 3YSZ supports with a 8YSZ porous interlayer (15 µm) and a NiO-8YSZ anode of 25 µm (with 31% porosity in oxidized state) [127]. RedOx cycles were performed during 30 min under air at 800 °C. The impedance spectra were fitted with an equivalent circuit based on a resistance (*R_s_*, ohmic resistance) in series with three RC processes of a resistance and a constant phase element in parallel. This gives three semi-circles of low (0.4–0.8 Hz), intermediate (6.2–10.9 Hz) and high (330–590 Hz) frequency phenomena. Ohmic resistance is constant while the polarization resistances at high and low frequency increase with RedOx cycling. The high frequency response is related to charge transfer and the low frequency response to gas diffusion and conversion. The authors explained the peak increase by the densification and the deterioration of the anode microstructure. 

Müller presented the evolution of the imaginary part of impedance versus frequencies after RedOx cycles at 950 °C [16] (Figure 26). In general, he observed an increase in the peak around 1 kHz and in some cases a slight increase in the low frequency peak around 1 Hz.

All studies on ESC Ni-YSZ revealed an increase of the complex impedance around 1 kHz during RedOx cycling. This is linked to the charge transfer at the Ni-YSZ anode active sites; an increase in complex impedance means a deterioration of the microstructure at the anode/electrolyte interface. 

#### 2.4.12. Single Chamber SOFC

In the case of a single chamber SOFC, the problem is different as both reducing and oxidizing gases are introduced together in the fuel cell. The specific activities of the two electrodes will produce a potential difference and generate current. Jaques-Bedard *et al.* observed an oscillation of the potential of a Ni-YSZ anode supported cell under a methane-to-oxygen ratio (M/O) lower than 2. This oscillation with a period of 20 s is related to the reduction and oxidation ongoing at the nickel surface. The degradation is more elevated for M/O < 2 and is explained by higher Ni evaporation at the fuel entrance and damages due to RedOx cycles [140]. Similar tests were done adding anode resistivity measurements. A voltage decrease was correlated to the anode resistivity increase; it was concluded that reduction-oxidation of Ni in the anode induced the voltage oscillation [141].

To clarify the effect of flowing both reducing and oxidizing gases over the anode, Kellogg *et al.* studied the Ni-YSZ anode in a double chamber, electrolyte-supported cell configuration. They flew 2/3 H_2_ and 1/3 O_2_ diluted in 95% of Ar over the anode, and pure oxidizer gas over the cathode. Oscillations of the open circuit voltage were observed around the equilibrium voltage of NiO/Ni at 600 °C. The explanation is an oxidation of the nickel and a re-reduction due to accumulation of H_2_ (the period is about 70 s in this case). Electrical conductivity measurements under these conditions showed a similar oscillation. When the H_2_/O_2_ mixture was flown over the cathode (with reducing gas over the anode) no variation was observed [142].

### 2.5. Summary of the RedOx Instability

One of the main limitations of nickel-based SOFC anodes is its RedOx cycling instability. The RedOx instability is coming from the volume change of nickel between its reduced and oxidized states. The volume increase during nickel oxidation induces an expansion of the composite. This expansion has three origins: (1) The reorganization of the metallic nickel during utilization; (2) the fast oxidation kinetics of the nickel at the operating temperature (between 600 and 800 °C) and (3) the closed porosity formation during the oxidation process. At low temperature or high oxygen partial pressure, the oxidation-limiting factor is the solid-state diffusion (which is thermally activated) giving a homogeneous oxidation of the full anode layer. In opposition, at elevated temperature and low oxygen partial pressure, the oxidation-limiting factor is the O_2_ gas diffusion through the oxidized anode layer leading to an inhomogeneous oxidation and higher layer internal stresses. Increasing reoxidation temperature will increase the expansion of the anode and the damages to the ceramic network.

The anode-supported cell (ASC) configuration is the most sensitive cell design: an anode expansion of 0.2% already induces cracks in the thin electrolyte. For the electrolyte-supported cells (ESC), the expansion limit before delamination of the anode is increased to 0.5%. In the case of cells on inert supports (RedOx stable metal or ceramic support), the expansion limit is even higher (around 1%). 

Various causes might induce anode oxidation during operation: air leakage (lack of fuel, shutdown and start-up without reducing gas, compressive sealing), high current demand, and fuel starvation. In the last two cases, the anode will oxidize due to ionic current (O^2−^) coming via the electrolyte. With these kinds of RedOx cycles, only a low amount of oxidized nickel (small degree of oxidation) will cause damages to the cell. 

After a RedOx cycle, the electrochemical performance of a cell might either decrease or increase depending on the severity of the cycle. The ohmic resistance can decrease after a RedOx cycle as shown by the electrical conductivity increase, but the degradation is often accelerated due to ceramic network damages. Concerning the polarization resistance (*R_p_*), if the RedOx conditions are severe (high temperature), as is normally the case for ECS, the ceramic network suffers and a decrease in performance is measured due to a decrease in triple phase boundary (TPB) length. On the opposite, if the RedOx cycle conditions are soft (RedOx cycle at low temperature, 650 °C), changes in the nickel morphology may induce even an increase in the TPB length and hence a lowering of *R_p_*. In case of an ASC, if the thin electrolyte cracks severely, the cell is destroyed. If the cracks are not too severe, the open circuit voltage will drop but the local temperature increase can nevertheless lead to enhanced performances under low fuel utilization. 

In general, published results do show a relatively large scatter which can be attributed to the variation of (1) the microstructure, including particles size and porosity (parameters not always given in the literature); (2) the composition of the sample; (3) the testing procedure and setup (duration and oxygen partial pressure) and (4) the design of the cell (including the active functional layer, the interlayer and the contact layer of the anode). 

Directions to improve the RedOx stability of the anode can be suggested from the results reported in this chapter. The next chapter will present and organize published solutions. They are separated in two families: (1) Solutions coming from the system itself and (2) solutions based on variations of the cell and its materials. Unfortunately not all solutions are precisely presented in the literature, especially those based on a patent. On the opposite, some alternatives, such as on ceramic anodes, are so largely described that they could be a subject for a review on their own. 

## 3. RedOx Solutions

**Figure 22:**
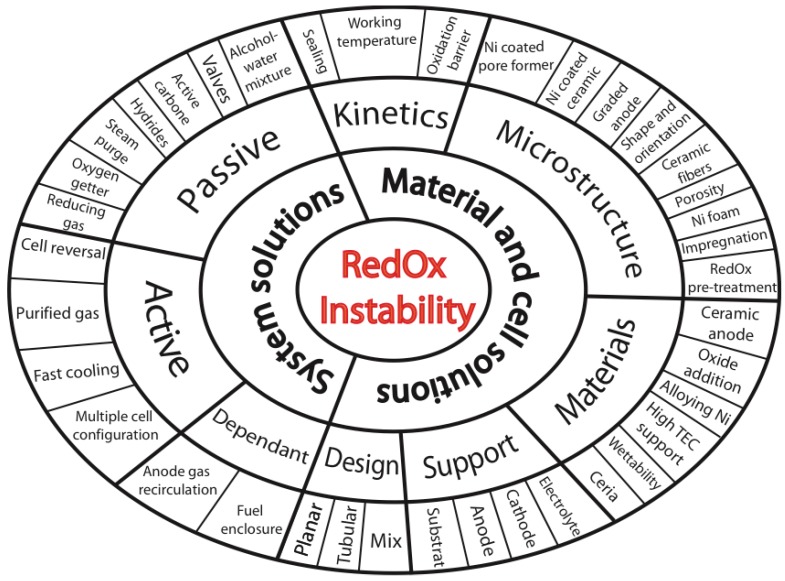
Summary of the solutions for anode RedOx instability [129].

This chapter attempts to give a complete overview of the RedOx solutions reported until now in the scientific community and in patents. A review has been made by Wood *et al.* from Versa Power Systems Ltd. (VPS) for small-scale residential and industrial power generation (3–10 kW_e_) based on anode-supported cells [143,144]. The potential solutions can be divided in two general families as summarized in Figure 22:

1. **System solutions** aim at keeping an oxygen partial pressure low enough to protect the anode from oxidation based on the global balance of plant of the SOFC system. They have two challenges: (1) compensate the RedOx limitation of anode materials; (2) include safety implementation of the anode and fuel processing gas. Hydrogen mixtures can explode below the autoignition temperature and carbon monoxide can be dangerous because of its flammability, toxicity and its propensity to react with nickel at a temperature below 230 °C to form the volatile and toxic nickel carbonyl.
  System solutions must protect the whole stack under normal events including varying power output, start-up and shutdown. But the unusual events are more dangerous for the stack, such as (1) system shutdown without fuel but with power available, and (2) emergency stop of the system (“blind shutdown”), without fuel and power.
  System solutions are grouped into dependent (to the system design), passive (no electrical power needed) and active (requiring the use of electrical power) measures.
  For small stacks (<1 kWe), system solutions are too expensive and good alternative ways must be found in the second approach.
2. **Materials, cell and stack design solutions**, such as alternative anode materials or optimization of the anode composition and microstructure. This approach is clearly passive and its cost is likely to be minimal. Therefore, while giving a brief overview of system solutions, this review will focus more on materials and design solutions.

A summary of the RedOx cycle degradation measurement as a function of the different solutions, especially from the second group, is presented in Table A1 (Appendix).

### 3.1. System Solutions

#### 3.1.1. Dependent System Solutions

In the **anode gas recirculation**, the anode atmosphere will stay longer in a reducing environment upon fuel supply interruption; only about 10% to 15% of the anode gas mixture would be lost and would need to be changed (by a reducing or neutral gas).

The **fuel enclosure** solution is similar to the “anode recirculation” solution but with a closed circuit, hence 100% of the gas is recirculated.

#### 3.1.2. Passive System Solutions

**Metal hydrides** trap hydrogen within an alloy. It is the best technology of a hydrogen container. When heat is applied, the gas is released. Reversible materials such as magnesium hydrides (MgH_2_) are typically used at 300 °C. At 650–700 °C no material is known to possess the same properties and more investigations are needed, but the high (endothermal) enthalpies of formation of these materials do not seem to be an issue for the SOFC protection. Mukerjee *et al.* describe some candidate materials [145].

**Reversible oxygen getter and sacrificial materials** solutions use unstable materials, such as nickel itself, to chemically react with any free oxygen that enters into the anode vicinity at high temperature. It would keep the oxygen partial pressure in the anode side low or the potential of the anode in a region where there is no oxidation of anode active materials. This approach is presented by England *et al.* [146] and Haltiner *et al.* [147] from Delphi company.

Assuming that liquid water is available, it can be added to the system and vaporized by the thermal energy contained in the hot balance of the SOFC plant. The reformer can be used to set up a slightly reducing gas by oxidizing some of the reforming catalyst as shown for nickel in Equation (1) (from right to left). As an alternative to the reformer, an additional nickel containing bed can be used. This **steam purge** can reduce the cell degradation by a factor higher than 30 [143,144].

**Valves** can be closed by gravity when the partial pressure of oxygen increases to a threshold value. This can be coupled with the use of an oxygen getter [147]. Using ceramic-glass sealing, the emergency valves closing can protect the stack during 15 h at 750 °C, which gives enough time for stack cooling [143,144].

By having an **alcohol-water mixture**, the fuel composition is adjusted to give a desired purge gas. The idea is to use the thermal energy contained in a hot SOFC to drive the endothermic reforming of the fuel (ethanol) (Equation (9)) [148].


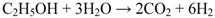
(9)

**Thermal cracking** of a stored fuel source uses the same idea as before but uses the cracking reaction instead (Equation (10)).


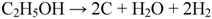
(10)

The **activated carbon** solution approach uses a carbon bed, which is an irreversible solution because at high temperature, air oxidizes the carbon to produce carbon monoxide and carbon dioxide gas [145].

#### 3.1.3. Active System Solutions

The use of a **partial oxidation reformer** can generate a suitable reducing gas for anode protection [149]. 

As explained in Section 2.2.2, the cell voltage is directly related to the gas composition at the anode and cathode through the Nernst potential. Thus, if an external voltage is applied to a cell or stack, this **cell reversal** is expected to reverse the flow of oxygen ions to maintain the anode at a safe oxygen partial pressure and protect the oxidation of the metal. The theory predicts this protection voltage on a simple gas/metal oxide/metal system but the cermet mixture makes it different [47]. This concept is outlined by Mukerjee *et al.* from Delphi company [145]. Recently, Young *et al.* studied the application of a constant cathodic current or potential to the anode during RedOx cycles. An ESC with a Pt counter, a Pt reference and a NiO-8YSZ (56–44 wt %) working electrode (0.4 cm^2^) was tested with constant potential or current of −150 mV, −350 mV, −6.5 mA and −17.5 mA under humidified hydrogen and air for 20 to 80 min at 800 °C. Main results are that under potentiostatic mode, the ASR decreased in opposition to galvanostatic mode where the ASR increased [150]. Further investigations, in particular on the microstructure, should be done to understand these results.

The **independence of cells** would allow changing the current density for each cell. It could also use the working cell in a similar manner as explained in the previous paragraph. But in case of a fuel supply problem, this approach is limited. This idea was presented by Backhaus-Ricoult *et al.*, who showed an activation during 2 RedOx cycles at 720 °C without indication on the anode composition [151].

As the kinetics are strongly dependent on temperature, **fast cooling** of the stack with a rate of 3 °C min^−1^ or higher (between 800 and 600 °C) will slow down sufficiently the kinetics so that standard Ni-YSZ anode support cells can withstand the oxidation [152].

Treating the air with a purification device to separate the oxygen and the nitrogen, the nitrogen is flown to the anode compartment and oxygen enriched air at the cathode side. **Purified air** can protect the anode against reoxidation during cooling down [153].

### 3.2. Stack Design

#### 3.2.1. Planar Design

The planar design is the most studied one because it can reach higher power volume density. The design of such a stack can be optimized to limit reoxidation of the anode supported cell. Van herle *et al.* calculated the partial pressure of oxygen depending on the fuel utilization (*F_u_*) for an open post-combustion design with counter flow, the so-called R-design. The reoxidation of Ni was obtained already at *F_u_* of only 64% [33].

In a similar manner using computational fluid dynamics (CFD) modeling of the partial pressure of oxygen, Larrain *et al.* calculated the risk of oxidation versus the *F_u_*. For a counter-flow configuration, the *F_u_* limit is given as a function of temperature and hydrogen flow rate. At 710 °C under adiabatic conditions the maximum *F_u_* is decreasing with increasing fuel flow rate from 92% at 200 mL/min to 89% at 400 mL/min. For the co-flow case, the limitation is only determined by the total fuel utilization [11]. 

Implementing the leakage in a compressive seal in the CFD model, Wuillemin *et al.* showed that high *F_u_* would decrease the active cell area. Using mica as sealing in an R-design configuration, the reoxidation of the active zone starts at 30% *F_u_*; at 68% *F_u_* the decrease in active zone is about 1.7% [154]. Based on CFD modeling, the flow design of the planar cell was optimized to limit the reoxidation of the cell [155]. 

#### 3.2.2. Tubular Design

This design can be seal-less and is known to resist transients [20]. The University of Birmingham studied the behavior of tubular anode supported cells with 200 µm anode thickness, 15 µm electrolyte thickness and with 2 mm of external diameter (produced by co-extrusion by Adaptative Materials Incorporated, USA) [20,156]. The electrochemical degradation and linear expansion were studied against temperature (at 600, 700 and 800 °C) and oxidation time of RedOx cycles (5 min and full RedOx cycle). The degradation increases with increased temperatures after a full RedOx cycle. The cell no longer worked despite relatively small expansion (see Table 5). Other studies also showed high degradation of cell performances under RedOx cycles [104]. 

Anode-supports with 10 mm of diameter showed high degradation as well (strength, conductivity, electrochemical) after 8 h RedOx cycles at 800 °C [157].

As the tubular cell can withstand relatively high cooling rates, an optimal cooling rate should be found to limit the degradation of the RedOx cycle by slowing the kinetics without increasing the degradation due to thermal shock [158].

In all these studies, the electrolyte is deposited exterior of the anode support. A small expansion of the support will then create large tensile stresses in this layer. If the electrolyte was deposited inside the support, then it could even be under compressive stress if the anode expands. This might be a solution for the tubular design. 

The **mixed design** aims to combine advantages of the seal-less tubular design with the high volume density of the planar design [159].

**Table 5:** Influence of temperature and time of reoxidation on the electrochemical performance and the linear expansion of tubular anode supported cells [20].

T/°C	52 RedOx cycles of 5 min, ∆*i*/*i* at 0.5 V	After first full oxidation cycle, ∆*i*/*i* at 0.5 V	Expansion during first full oxidation	Time to full oxidation (h)
600	−0.38%/cycle	−35%	0.20%	4.5
700	−0.42%/cycle	−61%	0.33%	3.0
800	−0.44%/cycle	−72%	0.46%	0.5

### 3.3. Cell Design

#### 3.3.1. Cathode Supported Cell (CSC)

The cathode supported cell was used in Siemens-Westinghouse technology and showed very long operating times (>40,000 h) with low degradation, but no mention on RedOx cycling was given [1]. The main drawback of the Siemens-Westinghouse technology is the elevated price of cell production. Huang *et al.* presented an electrochemical activation after 2 RedOx cycles for a 1 mm LSM porous support with a YSZ electrolyte and a noble metal anode based on Pd (1 µm median size) and YSZ (0.17 µm median size). At 800 °C, the power density was 0.15 Wcm^−2^ at 0.5 V [160]. No mechanical model describes this configuration under a RedOx cycle. 

#### 3.3.2. Electrolyte Supported Cell (ESC)

The electrolyte supported cell is a robust cell under RedOx conditions [106], but due to the high ohmic loss in the thick electrolyte at low temperature (700–800 °C), higher temperature must be used that makes the impact of the faster reoxidation important even for this cell configuration. 

ESCs under RedOx treatments have been well studied especially by Hexis and Kerafol [161,162]. In 2004, Hexis proposed to add doping elements to the nickel oxide to increase RedOx stability. They then showed 40% performance degradation over 3 RedOx cycles [161]. Four years later, the degradation of a 5-cell stack (with 120 mm diameter cells) over 11 RedOx cycles was lowered to 24% of area specific resistance (ASR) increase. Button cells showed about 40% degradation over 50 RedOx cycles (the first 30 RedOx cycles presented only small degradation) [163]. By changing the electrolyte composition from 8YSZ to 10ScSZ, a decrease of 50 °C in operating temperature could be brought about maintaining similar performance [164]. This also enhances the RedOx stability of the cells: a 5 cell-stack did not show any degradation over 12 RedOx cycles at 900 °C but then lost about 160 mV under constant current density for the last 8 RedOx cycles. A full system worked for 15,000 h with 4 thermo-RedOx cycles and 3 RedOx cycles with 1.9%/kh degradation. Further optimization work on the anode composition showed that the Ni-8YSZ is better at 950 °C under RedOx conditions compared to Ni-40CGO, due to better electrical conductivity. At 850 °C the effect is reversed and the Ni-40CGO is more stable due to a constant polarization resistance [81]. The Ni-40CGO thin anode was studied with *in situ* X-ray diffraction, the time for full oxidation of the nickel in the anode is 4 min at 650 °C and only 0.5 s at 850 °C [64]. Development on the anode microstructure and composition showed that coarse NiO-YSZ maintains a high conductivity under RedOx cycling and 40:60 vol % Ni:YSZ composition is more stable than 35:65 vol % [126] .

In parallel, Kerafol observed a constant ASR for 3 RedOx cycles at 850 °C using a 10Sc1CeSZ electrolyte and a Ni-8YSZ anode. During 3 further RedOx cycles, the ohmic and anodic polarization resistances increased [162]. The total ASR increased from 0.37 to 0.47 Ohm cm^2^ after 6 RedOx cycles. The microstructural analysis showed that the porosity of the tested cell had increased strongly. They observed a large scattering of the measurements because the RedOx cycles influenced also the contacting of the anode [131]. Microstructure optimization showed that coarse NiO (keeping the same YSZ) enhances the RedOx stability: After 10 RedOx cycles of more than 3 h at 850 °C, cell performances stayed stable at 0.7 A cm^−2^ at 0.7 V [165].

More recently, Staxera GmbH reported a study on 30 cell-stacks of 3YSZ thick electrolyte, Ni-CGO anode and LSM-YSZ cathode (128 cm^2^ active surface). After 80 thermo-RedOx cycles (cooling down without protective gas from 850 °C at a rate of 100 °C/h), the power output decreased by about 10% (0.125% power degradation per cycle). By changing the reduction conditions from 30 min at 700 °C to 5 min at 800 °C, the degradation per cycle increased to 0.44%. Pure RedOx cycles (20 min air flushing at the anode at 800 °C) degraded the stack power output of 1% per cycle [166].

Ouweltjes *et al.* tested a 25 cm^2^ electrolyte supported cell (ECS) of 3YSZ with an AFL of 80 wt % of 10CGO and 20 wt % of infiltrated NiO. The anode-contacting layer was based on La_0.9_Mn_0.8_Ni_0.2_O_3_ 30 wt % and 70 wt % Ni. This cell was RedOx-cycled for 120 min in air at 850 °C. A degradation of 10% was measured after 50 cycles and 24% after 100 cycles. Performances started at 0.36 A/cm^2^ at 0.7 V and ended at 0.28 A/cm^2^ at 850 °C, with 50% H_2_–50% H_2_O as fuel mixture [167].

Ukai *et al.* proposed to enhance the strength of the electrolyte by adding 0.5%–5% of Al_2_O_3_ to 3–6 mol % ScSZ to obtain RedOx stable cells. They claimed to achieve constant OCV and performance after RedOx cycles at 950 °C [168].

Weber compared ESCs with ASCs configuration over 50 short and 50 long RedOx cycles (applied successively); during the long RedOx cycle, the OCV dropped to zero for all ASCs and the performance degraded rapidly for all ESCs [169].

#### 3.3.3. Metal Supported Cell (MSC)

This cell configuration should be the most stable under RedOx conditions as shown in Section 2.4.6 [106]. 

The configuration can be made as (1) Substrate/Anode/Electrolyte/Cathode (S/A/E/C) [170] or reversed with (2) S/C/E/A [171]. Usually, due to cost limitations, an iron-based support is chosen. The benefits with configuration (1) are more freedom on the cathode composition. The major limitation is interdiffusion of Fe (from the metallic support) and active Ni (from the anode). A solution for a stable active anode is to impregnate the support with ceria or other salts after the electrolyte densification [170]. The benefits of configuration (2) are (a) no interdiffusion of Ni/Fe; (b) less corrosive cathode atmosphere for the metal support and (c) more freedom in the anode composition. One limitation is however chromium poisoning of the cathode; the composition should be tuned to limit this degradation.

The German Aerospace Center (DLR) SOFC technology is based on plasma sprayed layers on a metal substrate. In 2004, they showed a CroFer22APU porous structure with a Ni-YSZ anode under 7 RedOx cycles (40 min of oxidation at 800 °C) with an electrical conductivity decrease from 2200 to 2000 S/cm during the first 3 RedOx cycles, which then stays constant. During these cycles, both OCV and performance remained constant (1.03 V, and 400 mW/cm^2^ at 0.7 V 800 °C) [172]. Three years later, 10 RedOx cycles could be performed with only small OCV decrease from 1.049 to 1.037 V (0.11% degradation per cycle). The performance stayed stable around 149 mW/cm^2^ at 800 °C and about 0.75 V [173]. After optimization of the layers, more than 20 full RedOx cycles were performed on 12.5 cm^2^ cells without measurable degradation in OCV and less than 2.5% degradation in power density [174]. A stack of two cells with 82 cm^2^ active surface each, tested over 20 RedOx cycles at 800 °C during 1 h under pure oxygen, showed an increase in cell performance during the first five RedOx cycles after long period of 1250 h testing (for cell-1 from 128 to 156 mW/cm^2^ and cell-2 from 158 to 177 mW/cm^2^). After the 20 RedOx cycles, the performance of cell-1 and cell-2 degraded about 12.5% and 5.6%, respectively. This was attributed to increases in anode polarization resistance (+180%) and in electrolyte ohmic resistance (+50%) [133].

**Figure 23:**
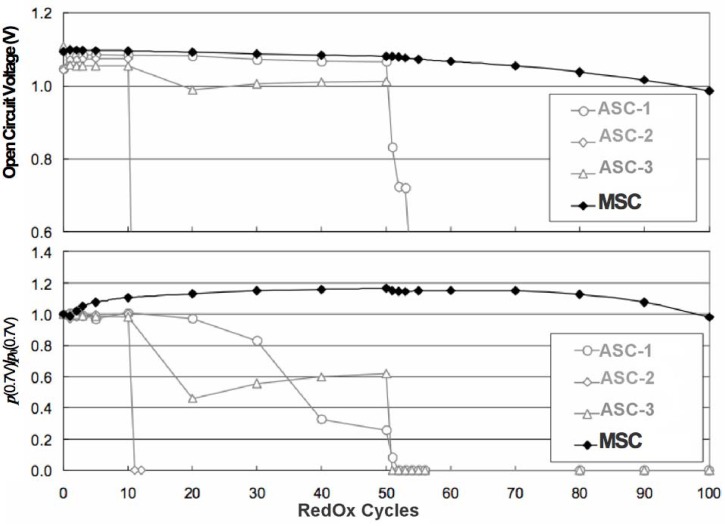
RedOx stability of anode supported cells (ASCs) (with support thickness of 1, 0.4 and 0.5 mm for ASC-1 (from Forschungszentrum Jülich), ASC-2 and ASC-2, respectively [14]) compared to metal supported cell (MSC), under 50 RedOx cycles of 1 min and 50 RedOx cycles of 10 min at 800 °C. Top: open circuit voltage, bottom: normalized performance at 0.7 V [175].

Metal supported cells produced by tape-casting with an anode of CrFe (350) or CrFe and YSZ were presented by Blennow *et al.* [175,176]. Sintering was done under reducing atmosphere with ScYSZ electrolyte, followed by infiltration with 20CGO with 10 wt % NiO and calcination for 2 h at 350 °C. The RedOx stability of these cells was compared to anode supported cells based on Ni-YSZ (with support thickness of 1, 0.4 and 0.5 mm for ASC1 (from FZJ), ASC2 and ASC3, respectively [14]) under 50 RedOx cycles of 1 min at 800 °C and 50 RedOx cycles of 10 min at 800 °C (see Figure 23). During the first 50 cycles, OCV was constant and performance slightly increased. During the next 50 cycles, OCV and performance decreased about 15% to 20%. A big difference could be observed between metal and Ni-YSZ anode supported cells.

A tubular metal supported cell (based on the configuration: Metal support/porous YSZ/dense YSZ/porous YSZ/metal support) was infiltrated by LSM (twice) and Ni (10 times). After each infiltration, the cell had to be fired at 650 °C. After 5 RedOx cycles, 26% degradation was observed with an initial power density of 650 mW/cm^2^ at 0.7 V at 700 °C under H_2_ and pure O_2_. For long-term stability, the Ni needs to be pre-coarsened at 800 °C, giving performance of 80 mW/cm^2^ at 700 °C [177].

These results of RedOx stability of metal supported cells make them a very interesting technology for the future. Long-term stability of more than 10,000 h still remains to be confirmed.

#### 3.3.4. Inert Substrate Supported Cells (ISSC)

**Figure 24:**
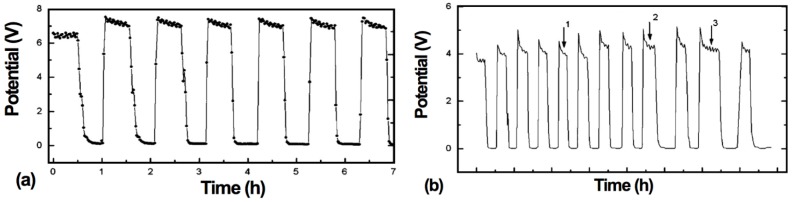
Voltage with time during RedOx cycles 800 °C of segmented-in-series (SIS) cells on a flattened partially stabilized zirconia tube support (under fuel, *i* = 0.9 A/cm^2^). In the first part of the test shown in (**a**), 12 SIS cells on one side of the module were tested during 7 cycles. The module was cycled to room temperature and then back to 800 °C before the second part of the test (**b**), where 9 SIS cells on the other side of the module were tested during 12 cycles. Arrows 1 and 2 indicate when the module was left overnight at 800 °C in hydrogen without cycling. Arrow 3 indicates a longer-than-usual (1 h) fuel feed [179].

**Figure 25:**
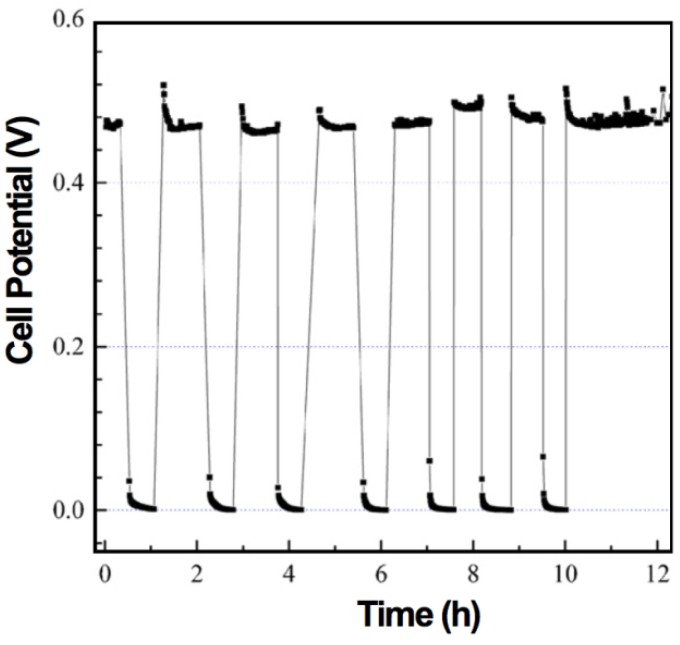
Time dependence of cell voltage of a Sr_0.8_La_0.2_TiO_3_ supported-cell with Ni-Ce_0.8_Sm_0.2_O_2_, Ni-YSZ anode and YSZ thin electrolyte over 7 RedOx cycles at 800 °C (under fuel, *i* = 0.9 A/cm^2^). The cell is alternatively exposed to dry H_2_ for 45 min and air for 30 min [180].

An inert non-conductive substrate of Ni-doped MgO-YSZ composite was used by Tokyo Gas for segmented-in-series (SIS) cells with Ni-YSZ anode. Two RedOx cycle procedures were used: (1) start-up and cool-down under air/water vapor (a/w) ratio of 0.5 and (2) 1.5 for 30 min at 775 °C under similar a/w. The open-circuit voltage stayed constant and the electrochemical performances decreased a few percent after 20 RedOx cycles of type 1 and isothermal cycles (type 2) [178]. Another study showed a SIS cell based on a flattened partially stabilized zirconia tube with a 70:30 wt % NiO:YSZ anode, 8YSZ thin electrolyte and LSM-YSZ cathode. The RedOx cycles of 30 to 40 min under air at 800 °C were applied 19 times without noticeable electrochemical performance loss (see Figure 24, note that the OCV is not shown) [179]. Sr_0.8_La_0.2_TiO_3_ porous support coated with NiO-Ce_0.8_Sm_0.2_O_2_ (SDC), NiO-YSZ and YSZ thin electrolyte presented impressive performance (0.9 A/cm^2 ^at 0.5 V) and RedOx stability at 800 °C (see Figure 25) [180].

#### 3.3.5. Anode Supported Cell (ASC)

The anode supported cell technology is the most sensitive configuration to RedOx cycling but currently also the most popular one owing to high performance at low temperature thanks to the dense thin electrolyte. The next sections of this chapter will mostly describe the strategies used to enhance the RedOx stability of ASCs.

### 3.4. Modification of the Microstructure

In order to enhance anode redox resistance, anode microstructure evolution needs to be investigated under RedOx cycles. This sub-section reports the state-of-the-art.

#### 3.4.1. Anode Functional Layer, Anode Support and Anode Current Collecting Layer

Based on a mechanical model, it was shown that the most important goal is to limit the anode support (AS) expansion and in a second step to limit the anode functional layer (AFL) expansion (see Section 2.4.6) [109]. The composition of the two layers can be different: the microstructure of the anode support should be more porous to maximize the gas transport and should have a high electrical conductivity, whereas the AFL should be denser and finer to increase the electrochemical active sites (or triple phase boundaries). Waldbillig *et al.* separated the functions of the two layers and tested the structures for RedOx stability [41]. This approach was also followed by other authors [14,181,182]. 

**Figure 26:**
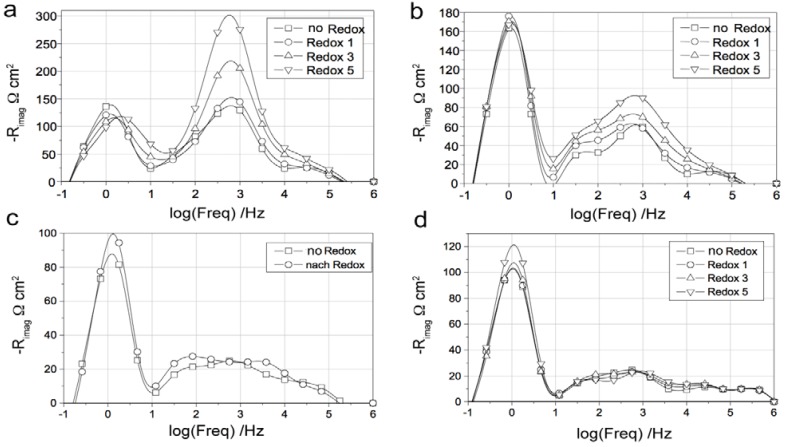
Electrochemical impedance spectroscopy at open circuit voltage (OCV), of electrolyte supported cell after RedOx treatments at 950 °C of sample composition (**a**) A; (**b**) B; (**c**) C and (**d**) D described in Table 6 [16].

In the electrolyte-supported case, Müller *et al*. separated the function of the AFL and the current collecting layer (CCL) and tested structures for RedOx stability [16,183]. He presented the evolution of the imaginary part of impedance versus frequencies after RedOx cycles [16]. He noted that the frequency peaks depend on the microstructure, the composition and the presence of a current collecting layer. Sample composition and electrochemical measurements after RedOx cycles at 950 °C are described in Table 6 and Figure 26. 

Without CCL, the imaginary part of impedance increases strongly around 1 kHz (Figure 26a). These frequencies correspond to the charge transfer process in the Ni-YSZ anode [136,137]. The cell after testing presented a large surface of total anode delamination (Figure 11 left). Adding a CCL on the active anode (sample B, Figure 26b) increases the low frequency peak (related to gas conversion and gas diffusion [134,135]). After RedOx cycles, the degradation at charge transfer frequencies is imitated even if the cell showed delamination between AFL and CCL (Figure 11right). With a finer AFL and sintered CCL (sample C, Figure 26c), both low (1 Hz) and high (1 kHz) frequencies were decreased. Changing NiO by Ni(OH)_2_ seems to enhance strongly the RedOx stability of the AFL (Figure 26d). This could be due to a difference in porosity of the active layer. 

**Table 6:** Composition and microstructure of the active and current collecting layer anode (the composition is always 65:35 mol % Ni:8YSZ for all layers and the active layer is sintered in all cases at 1350 °C) [16].

Sample	Active functional layer	Current collecting layer
A	NiO (1 µm)-8YSZ (0.8 µm)	None
B	NiO (1 µm)-8YSZ (0.8 µm)	Ni-8YSZ (no sintering)
C	NiO (0.5 µm)-8YSZ (0.5 µm)	NiO (0.5 µm)-8YSZ (0.5 µm), sintered at 1250 °C
D	Ni(OH)_2_ (0.5 µm)-8YSZ (0.5 µm)	NiO (0.5 µm)-8YSZ (0.5 µm), sintered at 1250 °C

#### 3.4.2. Particles Size

As early as 1996, Itoh *et al.* recognized the importance of the anode base material particles size for the stability of the cell. They showed (but only during a reduction reaction) that the use of a YSZ bimodal distribution (fine and coarse) led to a more stable anode [184]. More recently, Fouquet *et al.* measured the expansion of samples made of different NiO/YSZ particles size and sintered at 1300 °C. The expansions depended on the NiO/YSZ particle size ratio as follows: The lower expansion is for 0.5/0.2 followed by 0.5/0.8 and by 1.4/0.2, with particles size in µm. They found that the NiO particle size and the ratio between the particle size NiO/YSZ is the main factor for expansion during oxidation, but only three samples were tested [66].

Robert *et al.* changed the proportion of fine to coarse YSZ particles. They observed that the expansion is bigger in case of high content of fine YSZ [185].

Waldbillig *et al.* observed about 2.5% expansion for a fine microstructure and only 0.23% for a coarse microstructure during a RedOx cycle at 750 °C [41].

**Figure 27:**
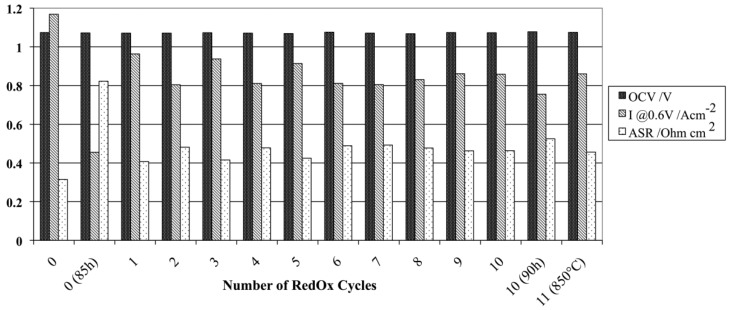
OCV, current density (*i*) at 0.6 V and area specific resistance (ASR) of anode-support containing 60 wt % fine NiO, 38 wt % coarse and 2 wt % fine YSZ with the number of RedOx cycles (the last cycle is done at 850 °C). Conditions: 97% H_2_ + 3% H_2_O at 800 °C. Measurement done 1 h after re-reduction when not stated otherwise [186].

**Figure 28:**
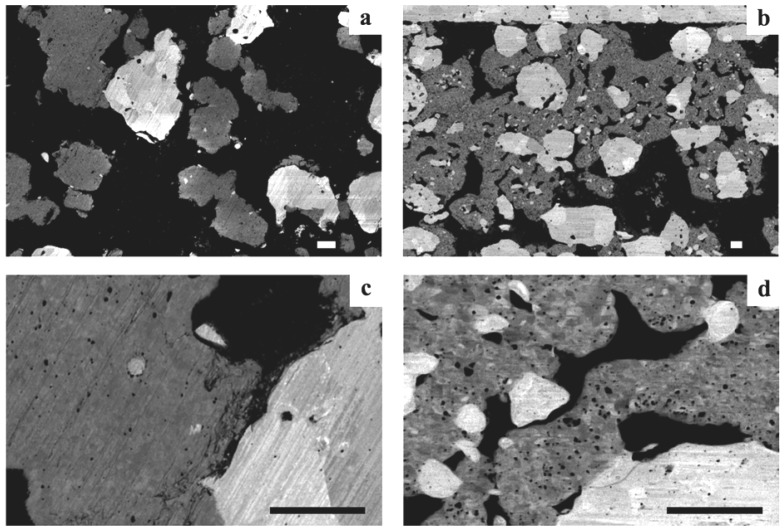
Anode containing 60 wt % NiO after 300 h at 800 °C under humidified forming gas (10% H_2_ in N_2_) (**a**) and (**c**): fresh as-sintered sample, and (**b**) and (**d**): tested sample from Figure 27. The grey levels separate each phase (YSZ: light grey, Ni: dark grey and porosity: black). All data-bars are 2 µm in length [186].

Design of experiment (DoE) approach was used to optimize the anode-support properties like RedOx stability, electrical conductivity and “surface quality”. The varied parameters were coarse and fine NiO and 8YSZ particles (with *d_V_*_50,*coarse*_ ≈ 9 µm and *d_V_*_50,*fine*_ ≈ 0.5 µm), composition (from 40 to 60 wt % NiO) and pore-former addition (from 0 to 30 wt %). Statistical analysis over 46 samples of 25 different compositions showed that the presence of coarse YSZ reduces RedOx expansion whereas changing the NiO particle size did not have a significant effect [86]. From the DoE study, three different anode-supported compositions (with 40, 50 and 60 wt % NiO) were tape-casted and tested during 10 RedOx cycles at 800 °C and one cycle at 850 °C. The OCV stayed constant over the cycles; the electrochemical performance dropped during the first utilization but was regenerated after a RedOx cycle and then stayed at the same value after multiple RedOx cycles (see Figure 27). The performance regeneration was believed to come from the creation of a porous Ni network stabilized by fine YSZ particles (see Figure 28) [186]. Scale-up of the cell size to 48 cm^2^ active area was performed and tested over 40 RedOx cycles at 800 °C. The OCV decrease by about 1 mV per RedOx cycle whereas the electrochemical performance stabilized after 4 RedOx cycles [187].

Conductivity measurements under RedOx cycling at 950 °C showed that coarse NiO-YSZ maintained high conductivity [126]. Microstructure optimization showed that coarse NiO (keeping the same YSZ) enhanced the RedOx stability: after 10 RedOx cycles of more than 3 h at 850 °C, cell performances were stable at 0.7 Acm^−2^ at 0.7 V [165].

Nickel carbonate pyrolized at 500 °C or 700 °C is composed of agglomerates (*d_V_*_50_ = 10–15 µm) of very fine NiO particles (surface area between 13 and 46 m^2^/g). Anode supports of such NiO mixed with fine standard NiO (*d_V_*_50_ = 0.5 µm) and 3YSZ or 4ScSZ gave good RedOx stability (analyzed by electrical conductivity measurement) compared to only standard fine NiO and zirconia composite. With only nickel carbonate pyrolized-zirconia composite, the electrical conductivity was however low before RedOx testing [188].

#### 3.4.3. Sintering Temperature

Lowering the sintering temperature from 1400 to 1100 °C seems to lower the damage in the YSZ skeleton and lowers the expansion from 0.6% to 0.1% after one RedOx cycle at 950 °C [66]. Robert *et al.* also noted a higher irreversible expansion for higher sinter temperatures, but did not notice any other influence [185]. 

#### 3.4.4. Porosity

**Figure 29:**
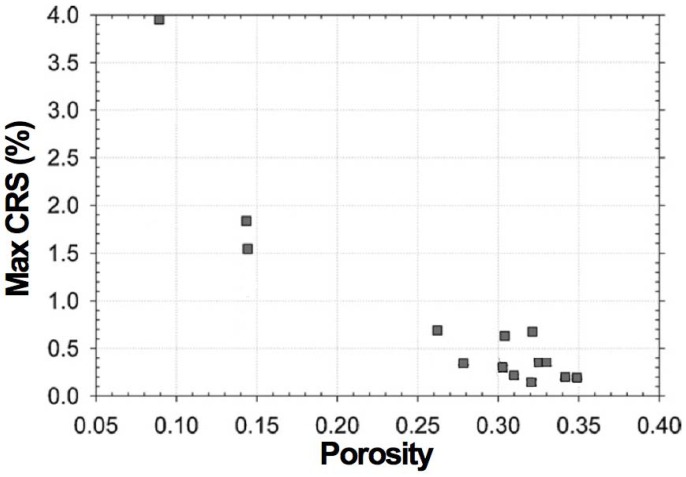
Maximum cumulative RedOx strain value (CRS) obtained after three isothermal cycles at 850 °C as a function of the total porosity of the Ni-YSZ composite [73].

**Figure 30:**
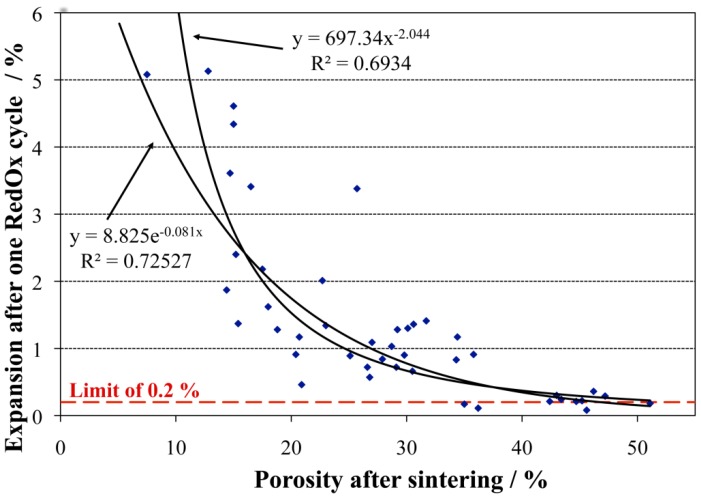
Expansion after one RedOx cycle at 800 °C versus porosity of 46 NiO-8YSZ anode-support samples [86].

Changing particle size and sintering temperature has a direct influence on the sample porosity. As the nickel expands to NiO, it is intuitive that an increase in porosity will let the nickel oxide fill the porosity without producing an expansion. This approach is proposed by Robert *et al.*, where an optimized microstructure containing macro- and micro-pores limit the expansion during RedOx cycles [189]. Pihlatie *et al*. observed that the increase in porosity decreased the expansion during RedOx cycles as shown in Figure 29 [73]. A similar observation was reported for 46 different NiO-YSZ samples. Porosity higher than 45% in the as-sintered state should give RedOx stable supports with an expansion limit lower than 0.2%, but some samples with only 35% porosity also present low expansion (see Figure 30) [86].

Inversely, Klemensø observed that low porosity is better for the RedOx stability as the strength of the support will be higher [27]. Ettler *et al.* showed that when varying the gas flow (from 20 to 1200 ml/min) for a constant temperature (800 °C) and a constant time of oxidation (15 min), the degree of oxidation (DoO) of an anode support depends on its porosity. Samples with 48% porosity showed a DoO reaching 100% and cracking of the thin electrolyte whereas a denser support with 33% porosity only reached about 20% of DoO and presented no crack in the thin electrolyte [14]. In this case, the limiting factor for oxidation is the gas diffusion process that is higher for the more porous sample. It should be noted that in practice, during air leakage or lack of fuel, the air flow will not vary so much but it will be more probable that time will vary and extend to longer periods.

#### 3.4.5. Composition

The proportion between Ni and YSZ was studied by dilatometry on a fine microstructure: lowering the NiO content (from 57, 40, 35 to 30 wt %) seems to decrease the linear expansion between 57 and 35 wt %, but the 30 wt % sample had again a similar expansion than the 57 wt % sample [41]. Inversely, based on coarse YSZ particle size, the expansion during RedOx cycles seems to be compensated by the large shrinkage during reduction for high NiO content samples [185].

From ESC electrochemical tests, 40:60 vol % Ni:YSZ composition appeared to be more stable than 35:65 vol % under RedOx cycling [126].

A statistical approach with 46 samples showed that the NiO content between 40 and 60 wt % does not have a significant effect on expansion during RedOx cycles [186]. This can explain the contradictory results reported by different studies in this range of composition. 

In fact, at low NiO content (<30 wt %), decreasing the nickel content would limit the expansion over a RedOx cycle as shown by Wang *et al.* They studied Ni-YSZ composite sintered 6 h at 1400 °C with low amount of Ni (0 to 30 vol % Ni) for non-conductive substrates for segmented-in-series cells. Dilatometry measurements showed a RedOx stable behavior for concentrations of 10 vol % Ni (equal to 17.6 wt % NiO) or lower [190].

In case of anode application, the electrical conductivity is essential. By lowering the nickel content, the electrical conductivity will dramatically decrease if the percolation threshold is violated (at 29.4 vol % for spherical particles with the same diameter [191]). Different strategies are proposed to decrease this threshold value.

#### 3.4.6. Orientation and Particle Shape of Nickel Phase

**Figure 31:**
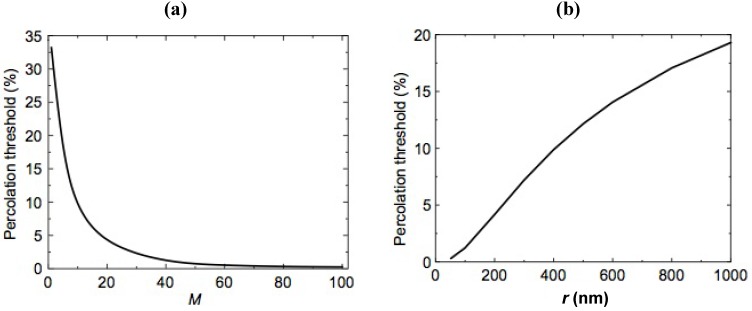
Percolation threshold versus (**a**) axial ratio *M* and (**b**) radius of conducting particles *r* [192].

The particle shape and size of the electronic conducting phase change the percolation threshold. According to Maxwell’s theory, the relation between conductivity and particle shape can be found. Using metal particles with a larger axial ratio (*M*) and a smaller radius (*r*) reduces the minimum metal content required to reach a certain conductivity value. Xue performed metal-polymer experiments in good agreement with theory, with an electric threshold around 5% of metal particles of *M* = 6 and ellipsoid semi-radii of 1651, 275 and 275 nm, respectively [192] (see Figure 31). This idea has been recently patented for the zirconia particles shape [193].

If the conducting phase could be organized, the percolation threshold could be decreased further. A way to organize the conducting phase is to put the tape-cast anode slurry in a magnetic field: as nickel and nickel oxide are ferromagnetic and antiferromagnetic, respectively, they will orient in the applied field [194]. Magnetism measurements can also give the proportion between Ni and NiO [195] and the average size of the magnetic particles [196,197].

#### 3.4.7. Ni coated Pore-Former

Graphite coated with Ni was used to produce Ni-YSZ anodes by tape-casting. The composite showed high conductivity with only 12 to 20 vol % of Ni (3–5 orders of magnitude higher than conventional anodes) [198,199,200,201,202].

#### 3.4.8. Ni Foam

Corbin *et al.* proposed to use nickel foam impregnated with a mixture of Ni, YSZ and starch pore former in a polyvinyl alcohol solution. The samples were then sintered at 1475 °C in air for 1 h and finally reduced in dilute hydrogen at 1000 °C for 2 h [203,204]. The electrical conductivity at room temperature shows that only 2 to 5 vol % of Ni (from the total volume including porosity) is needed to obtain more than 1000 S/cm compared to 10 vol % in the case of Ni coated graphite [202].

Ni porous structure impregnated with Fe was used as a support for a complex cell using a La-doped ceria-Ni composite thin anode. A single RedOx cycle of 2 h at 700 °C presented a performance increase of about 1% at 0.27 W/cm^2^ [205,206].

#### 3.4.9. Wet Impregnation (WI)

Wet impregnation uses dilute salts of active materials, which can be deposited inside a porous structure by a subsequent heat treatment, removing the organic material. This technique can be used to lower the quantity of expensive element or to avoid reaction between unstable components during sintering. A review on wet impregnation for SOFC application is compiled by Jiang [207]. The limitation of this technique is the low mass loading per cycle, which means multiple thermal treatments are needed to obtain sufficient material. This represents a problem for upscaling the process.

Jasinski *et al.* proposed to impregnate porous Sm_0.2_Ce_0.8_O_2_ produced by dry mixing with carbon powder (90:10 vol %). The porous substrate was impregnated with Ni nitrate to reach 7.5, 11 and 14 vol % Ni. The conductivity was tested over 10 RedOx cycles and seems stable around 80 S/cm for 11 vol % Ni. Measurement over time showed that the 14 vol % Ni reaches 80 S/cm after 100 h without change after a RedOx cycle [208].

A similar approach was to infiltrate a porous YSZ skeleton by Ni nitrate salt (10 times with decomposition at 500 °C to obtain 12–16 wt % Ni). No expansion could be observed upon one RedOx cycle of 100 min at 800 °C. The room temperature conductivity is 360 S/cm and 290 S/cm after the RedOx cycle [117].

Zhu *et al.* reported a RedOx stable YSZ skeleton support impregnated with nitrate solution including La^3+^, Sr^2+^, Cr^3+^, Fe^3+^ and Ni^2+^ ions and urea. The final impregnate anode is about 35 wt % LSCF-oxide and 5 wt % Ni. The cell gave stable performance of 0.5 A/cm^2^ at 0.42 V and 800 °C after 10 RedOx cycles between p_O2_ = 0.3 and a CH_4_/O_2_ ratio equal to 2.2 [209].

Buyukaksoy *et al.* reported an electrolyte-supported cell with a 10 µm porous YSZ (sintered at 1150 °C) impregnated with nickel salt solution (loading about 30 vol % NiO after 20 cycles). The cell showed an activation upon the first 15 RedOx cycles at 800 °C, going from 0.8 to 1.5 A cm^−2^ at short circuit [210].

#### 3.4.10. Ni Coated Ceramic

Impregnation was used to coat fine (0.3 µm) and coarse (10 µm) YSZ particles with NiO (40:60 vol % Ni:YSZ). The powders were uniaxially pressed as discs of 1.2 mm thickness. Thermo-RedOx cycles up to 800 °C under air were applied to the samples. Electrical conductivity decreased from 1450 to 1250 S/cm over 20 RedOx cycles, whereas for standard NiO-YSZ composites, the conductivity decreased from 1200 to 600 S/cm for the same treatment (due to higher Ni coarsening). The coated particles were used to produce anode supported cells with an electrochemical performance of 0.56 W/cm^2^ at 800 °C and 0.5 V [211].

A similar process was used to coat NiO on YSZ and CGO. Composites made of 35:65 wt % YSZ:NiO and CGO:NiO showed high strength of 241 MPa and 146 MPa and high electrical conductivity of 2890 and 2710 S/cm, respectively [212].

#### 3.4.11. Graded Composition and Porosity

##### (1) Anode functional layer

The use of a graded content of Ni and porosity in the AFL showed an improvement in the RedOx stability of the anode as Waldbillig *et al.* demonstrated in a recent paper [213]. The RedOx sensitivity of the cell after a full cycle for the graded AFL is only half that of the standard one. 

This approach is also considered by Bloom Energy^®^ on ESC technology but with a gradient along the cell length and the anode thickness. This study was based on anode graded composition of Ni and Ce_0.8_Sm_0.2_O_2_ (SDC) done by ink jet printing (higher content of SDC close to the entrance of the fuel and close to the anode-electrolyte interface). The cells were compared to standard cells in a 10 cells stack configuration. The oxidation was performed under constant temperature and constant current load by decreasing the fuel flow to zero in about 5 h. After the first RedOx cycle, the *R_s_* and *R_p_* were compared between the different cells, showing an *R_s_* increase of 24% for standard cells and only 3% for the new cells. The *R_p_* increased by 22% for standard cells and decreased by 1% for modified cells. After the second RedOx cycle, the overall *R_s_* increase for the new cells was 5% and 1% for *R_p_* [132].

##### (2) Anode support

**Figure 32:**
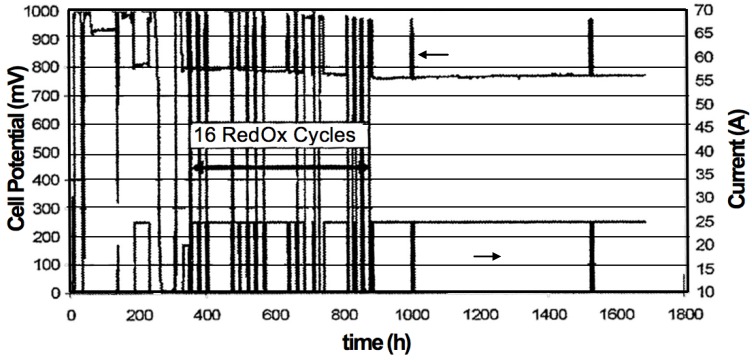
Anode supported cell tested over 16 RedOx cycles and 1700 h on a single repeat unit stack configuration. Test conditions: 800 °C, active surface area about 100 cm^2^ and constant current load of 0.25 A/cm^2^ [215].

A graded support was fabricated by slip casting using water-based slurry. The porosity gradient permitted to lower the porosity down to 30% without having too much diffusion limitation through the 0.8 mm thickness of the support. These supports could reach 39% electrical efficiency at 250 mW/cm^2^, 0.65 V and 850 °C in a Hexis stack. After a RedOx cycle at 920 °C, the OCV decreased from 990 to 850 mV, showing the limitation of this anode support at this temperature [90]. A similar approach was taken by Ihringer *et al.* who produced anode supports of thickness from 0.3 to 1.2 mm with lower amount of nickel (addition of starch pore former was used to increase porosity) [105]. The electrochemical tests on 1 cm^2^ gave initial power density of 0.98 W/cm^2^ and a final one of 0.7 W/cm^2^ after 10 RedOx cycles at 800 °C and 0.7 V. Repeat element configuration with a 44 cm^2^ active surface shows a constant OCV and a small decrease of potential from 0.89 to 0.87 V at 0.23 A/cm^2^ and 800 °C after 10 RedOx cycles, 2 thermal cycles and 400 h of utilization. The mechanical strength of these supports increases slightly from 145 to 155 MPa after 10 RedOx cycles (the Weibull modulus also increases from 6 to 9) [214]. Further studies showed 75% of *F_u_* and 42% of electrical efficiency (at 0.38 W/cm^2^, 0.7 V and 806 °C) [181]. A recent study on a single repeating unit stack of 100 cm^2^ active area (based on Hexis design) tested over 1700 h and 16 full RedOx cycles (more than one hour under air at 800 °C) gave a powder degradation (at 0.25 A/cm^2^) of 0.3% per cycle. The OCV dropped about 40 to 50 mV during the measurement (see Figure 32) [215].

#### 3.4.12. Controlled RedOx Cycle

As RedOx cycles change the sample microstructure, it was proposed to apply to the anode support a controlled RedOx cycle to enhance the RedOx stability. Wood *et al.* observed a lower decrease in performances on preconditioned samples (one RedOx at 550 °C) with only 3.2% decrease in voltage at 0.75 A/cm^2^ after a RedOx cycle at 750 °C compared to 10.8% decrease without the preconditioning. There are several stages in the fabrication where the initial controlled RedOx cycle may be applied. It can be done on the mixture prior to the formation of the green anode structure, then on the fired sample before insertion in the stack and finally, *in situ* in the stack [216]. 

Pihlatie *et al.* showed an increase of electrochemical performance after a RedOx cycle at 650 °C in a symmetrical cell configuration [73]. Different groups observed an increase in performance over short-term RedOx cycles [175,213]. This is believed to be due to the enhancement of the contacting layer at the anode side.

### 3.5. Alternative Anode Materials

#### 3.5.1. Alloys and Additives for Metal-Ceramic Anode

A potential solution is to use an alloy (or even noble metal) with higher oxidation resistance. The idea is to slow down the kinetics [145], make a protective layer on the nickel [145] or limit the nickel coarsening [189,217].

The addition of noble metal particles in the anode was presented by Huang *et al.* [160]. The idea was to cover the metallic phase by nanoparticles of oxide (YSZ, ScSZ, CGO, CeSmO, LSGM) to reduce vapor loss and agglomeration of noble metal particles. The proportion of the anode was around a third of each phase, metallic, ceramic and porosity. Some results are shown over two RedOx cycles with an ESC and CSC configuration, but the main limitation of the approach is the price of the noble metal.

Several authors alloyed copper with nickel [218,219,220,221,222]. The aim of Cu addition, usually coupled with a ceria-based ceramic, is more to limit hydrocarbon cracking than to enhance RedOx stability of the anode, but RedOx cycles have been tested at 750 °C under methane/air and shown slow regeneration over 100 h to reach initial performance [220].

Robert *et al.* proposed the addition of a doping element such as Al_2_O_3_, TiO_2_, CeO_2_, MgO or spinel compounds and salt or oxide from Ni, Mn, Fe, Co and Cu as sintering aids and MgO as inhibitor of nickel grain growth [189], to prevent RedOx instability of the Ni-YSZ anode support. The addition of CeO_2_ had already been proposed earlier by researchers from the Dornier Research Center [223,224] with apparently promising results but unfortunately very little published results.

Larsen *et al.* proposed to add another oxide to the anode (Cr_2_O_3_, TiO_2_, Sc_2_O_3_ , Al_2_O_3_, VO_x_, TaO_x_, MgCr_2_O_4_, CaO, MnO_x_, Bi_2_O_3_, LnO_x_ NbO_x_, …) [217]. The claim is to prevent nickel coarsening due to Ni-particle growth inhibitors, to surface passivate the Ni, to slow down the kinetics of oxidation and to strengthen the ceramic structure of the anode support and/or anode layer. The author tested the addition of 5 wt % of Cr_2_O_3_ in the anode support and observed formation of NiCr_2_O_4_ during sintering and the reduction of this phase resulted in a partial surface coverage of Ni particles that stabilizes the structure. The addition of 7 wt % TiO_2_ in the active anode layer forms NiTi_2_O_4_ that creates small particles of TiO_2_ after reduction of the anode. These particles prevent Ni particles from coarsening. The use of Cr_2_O_3_, TiO_2_, Sc_2_O_3_, Al_2_O_3_ decreases the anode thermal expansion coefficient.

Addition of equal molar amounts of NiTiO_3_ and (Sr,La)ZrO_3_ gives a composite of (Sr,La)TiO_3_, NiO and ZrO_2_ after sintering. After reduction the microstructure provides catalytic activity as well as electronic conductivity [217]. 

Jain *et al*. proposed to use a natural stone as sintering aid (Dolomite, D): CaCO_3 _+ MgCO_3_ + impurities (CaO 66.2%, MgO 32.3%, Al_2_O_3_, Na_2_O 0.34%, SiO_2_ 0.26%). This shows an increase in strength (maybe due to a decrease in porosity). Comparable electrochemical performances were presented with 2 wt % addition. No RedOx tests were shown in this study [225].

From alloy corrosion, it is known that a small addition of solute can increase the oxidation rate of Ni [46], but above a certain level (5 at %) a passive layer can be formed (e.g., of Cr_2_O_3_ or Al_2_O_3_,) depending on composition, which slows down or stops the oxidation [25] (see Section 2.2.2).

A kinetic study compared the addition of MgO, TiO_2_ and CaO (4 mol % in a 40:60 vol % YSZ:NiO) to a pure composite [36]. All dopants slow down the oxidation rate between 650 and 800 °C, the most efficient being CaO. The microstructures with dopant present less porosity, which could be due to a decrease of the Ni^2+^ diffusion along the NiO grain boundaries.

Another kinetic study based on thermogravimetric analysis (TGA) presents the addition of 1, 3, 5 and 10 mol % of Al and Ce to NiO (by nitrate salt addition). After homogenization with YSZ and sintering for 2 h at 1450 °C, the samples were crushed before being measured in the TGA. The results showed that the additives increased both the rate of reduction under dilute hydrogen and the rate of oxidation under air at 800 °C. Even if the additives are different, the results are not consistent with Tikekar’s study [36] but correlate better with the results on alloys [46]. This discrepancy could be linked to microstructural differences; grain boundary diffusion of Ni^2+^ should be lower but a decrease of the NiO grain size could again increase the oxidation rate. 

Expansion measurements showed that NiO doped with Ce (sintered around 1360 °C) presented maximal strain smaller than 0.1% during 3 RedOx cycles at 850 °C, compared to undoped NiO with a value between 0.2% and 0.3% [73]. Doping with Al_2_O_3_ gave 0.22% expansion. With MgO, the first RedOx cycle induced a significant expansion of 0.35%, the following cycles only 0.14%. Ceria could thus be an option to further lower the ASC expansion. Another study based on *in situ* curvature measurements on half-cells showed that 5 mol % of Ce in NiO bent the anode towards the electrolyte during reduction, due to the expansion of cerium in reducing atmosphere (cracks occurred in the thin electrolyte). Undoped samples bent towards the anode as usually observed [100]. Previous results reported by Klemensø presented an initial increase in length during reduction at 1000 °C of 0.7% for a composite based on 55 vol % NiO, 26 vol % 3YSZ and 19 vol % CeO_2_ [27]. The better additive is found to be 13.5 vol % of Al_2_O_3_ (with 51 vol % NiO and the rest 3YSZ), leading to 0.28% of strain during expansion at 1000 °C (compared to 0.35% for the undoped sample). Addition of 20 vol % TiO_2_ seems not beneficial, with more that 2% strain measured on a RedOx cycle. These two last studies are inconsistent with Pihlatie *et al.* as he observed a strong shrinkage (of 0.2%–0.3%) during reduction of the Ce doped samples [73]. The difference probably again stems from the difference in composition, microstructure and porosity of the samples. It is possible that by optimization the right proportion of NiO and CeO_2_ can be found, where the volume changes of the two phases can compensate each other during reduction and oxidation. 

In a different study, TiO_2_ seems beneficial against RedOx cycles [226]. Two compositions containing 1 wt % TiO_2_ were prepared as follows: (1) Nickel chloride hydrate and titanium tetrachloride (combustion method) mixed with 10Sc1CeSZ and (2) standard powder mixture of TiO_2_ and NiO. A RedOx cycle at 1000 °C in air resulted in a linear expansion of 0% and 0.04%, respectively (without TiO_2_: 0.34% and 0.31%, respectively).

A RedOx stability test at 800 °C was performed on Cu-LSCM (La_0.75_Sr_0.25_Cr_0.5_Mn_0.5_O_3−d_) pellets by four-point electrical conductivity measurement giving promising results [227].

(Mg,Ni)O (65:35 in mol %) solid solution showed an expansion under the first reduction of 30 h (of about 1%) and a shrinkage of 0.4% upon reoxidation [228]. The second reduction during 220 h induced a strong expansion of 3.5%. This is correlated to the exsolution of Ni separated to the grain boundaries of MgO (small particles of Ni were observed at the surface of MgO grains). This effect could be used to compensate the expansion of pure Ni during oxidation but Pihlatie *et al*. did not observe this effect adding a small amount of Mg to NiO [73].

Application of similar compounds was presented by Fujita *et al.* for a segmented-in-series SOFC substrate based on Ni-doped MgO with 8YSZ produced by extrusion. They showed partial RedOx cycles without electrochemical degradation [178]. Later, the same group compared the stress build up in NiO-YSZ and NiO-MgO-YSZ composite during RedOx cycles using the XRD technique; the compressive stresses in the thin YSZ electrolyte strongly decreased with NiO-YSZ whereas it stayed constant with MgO [229]. Dilatometry and residual stress in the electrolyte measurement showed a RedOx stable behavior at 800 °C of NiO-MgO-YSZ composite from 5 to 30 vol % NiO [230].

The addition of a stable oxide in nickel RedOx cycling was also studied for chemical looping combustion applications [231,232].

From this short overview on additives to Ni-YSZ anode for RedOx stability enhancement, it is not clear whether this strategy can be successful. The effects of these additives appear at any stage from fabrication to reduction and reoxidation. They will change the microstructure of the composite and of NiO by producing spinels like NiCr_2_O_4_ or NiAl_2_O_4_ and other compounds that can lower the sintering temperature.

#### 3.5.2. Full Ceramic Anode

This sub-section could be a full subject on its own. Here, only main results will be given. Ceramic anodes are considered to overcome the limitations of Ni based anodes, which are cracking of hydrocarbon fuel, poisoning with sulfur and other species, and limited RedOx stability. A general review for ceramic anodes is available in [233], another is specific on RedOx stability of ceramic based anodes [15].

The principal needs for ceramic anodes are [234]:

1. Negligible dimensional change during RedOx cycles (less than 0.1 to 0.2% of linear expansion).
2. Electrical conductivity higher than 10 S/cm.
3. Stability in reducing atmosphere and air and compatibility with the electrolyte.
4. Thermal expansion coefficient close to that of the electrolyte. In case of YSZ: between 10 and 11 × 10^−6^ K^−1^.
5. Good catalytic activity for H_2_ and CH_4_ oxidation. In case of mixed conductivity the ionic conductivity should be >0.02 S/cm.

Fu *et al.* proposed to separate the support from the active ceramic anode functions [15]. Where only the active layer must possess electrocatalytic activity and good ionic conductivity, the support needs high electrical conductivity. 

According to Table 7, the best candidates could be ZrTiYO_2_, LaSrCrMnO_3_, SrYTiO_3_ and LaSrTiO_3_.

**Table 7:** Summary of properties of some potential oxide anodes at 800 °C under reducing conditions [235].

Materials	CTE (10^−6^ K^−1^)	Electronic conductivity (S/cm)	Ionic conductivity (S/cm)	Polarization resistance	RedOx stability
CeO_2_	12	0.5–1	0.1–0.2	++	–
ZrTiY-oxide	10	0.1	0.01	+	++
LaSrCrRu-oxide	10	0.6	small	++	+
LaSrFeCr-oxide	12	0.5	?	++	+
LaSrCrMn-oxide	10	3	?	+++	++
LaSrCrV-oxide	10	?	?	++	++
SrYTi-oxide	11–12	80	small	+	+++
LaSrTi-oxide	10	40	small	++	+++
Nb_2_TiO_7_	1–2	200	very small	–	–
GdTiMoMn-oxide	?	0.1	reasonable	+	–
BaCe_0.8_Y_0.2_O_3_	?	0.02	?	–	++

The first paper on a full ceramic anode that shows RedOx stability was proposed in 1999 by Marina *et al.* The configuration was ESC of 180 µm thickness (8YSZ) and an anode of 8YSZ (anchoring layer made of coarse particles) and 40CGO sprayed 15 µm thick. A power density of 470 mW/cm^2^ was obtained at 1000 °C and 0.7 V in 9% H_2_. Several RedOx cycles were carried out by turning off the fuel gas and letting the OCV drop to 0 V without any degradation [236]. In a later study, La_x_Sr_1−x_TiO_3 _presented high electrical conductivity for samples sintered under H_2_. 14 RedOx cycles at 500 °C (overnight) caused a 40% decrease in conductivity, and then only 10 to 24 min per further RedOx cycle led to a final conductivity of 300 S/cm. The expansion was lower than 0.1% during RedOx cycles at 1000 °C. The good initial performance decreased rapidly after the RedOx cycles to only about 60 mA/cm^2^ [237,238].

Strontium titanates have been studied by several groups, because of their high electrical conductivity and dimensional stability under RedOx treatment. SrTiO_3_ doped with Y, Sc, La and cerium oxide doped with Nb, V, Sb and Ta were patented as ceramic anode to work in SOFC and solid oxide electrolyzer cell (SOEC) mode. The expansion during a RedOx cycle was lower than 0.1% [239,240]. A parallel patent proposed SrTiO_3_ doped with Y, La, Gd that shows expansion during RedOx cycles lower than 0.14% and a polarization resistance lower than 0.3 Ohm cm^2^ at 800 °C when infiltrated by Ni [241].

More recently Miller and Irvine proposed a study changing the B-site of La_0.33_Sr_0.67_Ti_0.98_**X_0.08_**O_3_ with **X** = Al^3+^, Ga^3+^, Fe^n+^, Mg^2+^, Mn^n+^, Sc^3+^. During TGA measurements, Mg showed the lowest amount of reoxidation strain in air up to 900 °C: +0.11% < Sc (0.14%) < Al (0.16%). Conductivity was higher for Ti (8 S/cm) > Al > Ga and the performances were better in case of Ti (0.32 A/cm^2^ at short circuit) > Mn (0.3 A/cm^2^) > Ga (0.26 A/cm^2^) > Al (0.25 A/cm^2^) [242].

Gross *et al.* developed a ceramic anode with high performance (850 mW/cm^2^ at 800 °C), composed of a thin active functional layer (AFL) on a non-catalytic conductive layer [243]. The AFL composition was 1 wt % Pd, 40 wt % ceria in YSZ. The support was based on La_0.3_Sr_0.7_TiO_3_ (LST).

La_0.2_Sr_0.8_Cr_0.8_Pd_0.2_O_3−d_-10GDC anode on LSGM (La_0.8_Sr_0.2_Ga_0.8_Mg_0.2_O_3−d_) electrolyte-support presented good electrochemical performance (0.47 W/cm^2^ at 0.6 A/cm^2^ and 800 °C) followed by 20% degradation over 200 h. After RedOx cycling at 800 °C, the performance regenerated probably due to Pd-nanoparticles re-nucleation during RedOx cycling [244].

**Figure 33:**
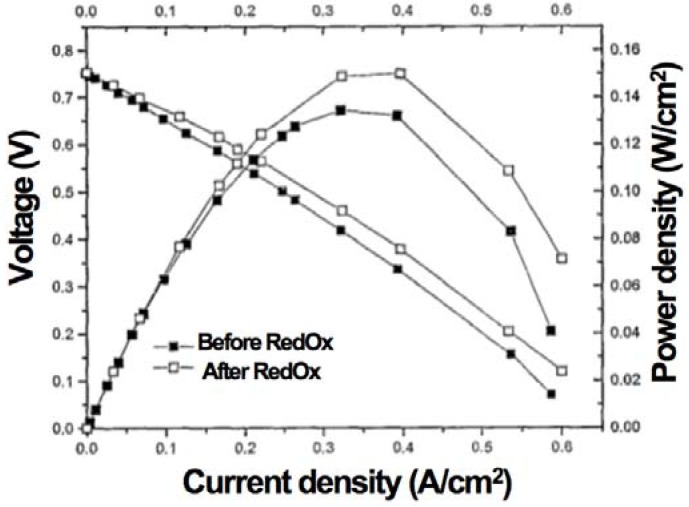
(La_0.75_Sr_0.25_)_1−x_Cr_0.5_Mn_0.5_O_3_ (LSCM) (47.5 wt %) + CGO (47.5 wt %) + NiO (5 wt %) under CH_4_ with 150 mW/cm^2^ at 750 °C before and after RedOx cycles [248].

Different papers propose the use of (La_0.75_Sr_0.25_)_1−x_Cr_0.5_Mn_0.5_O_3_ (LSCM) as anode and cathode materials for a RedOx-stable symmetrical SOFC [245,246,247]. Barnett *et al.* showed that LSCM (47.5 wt %) with CGO (47.5 wt %) and NiO (5 wt %) anode yields relatively good performance under CH_4_ with 150 mW/cm^2^ at 750 °C, activating during 4 RedOx cycles of 30 min under air (shown in Figure 33) [248]. Similar results are shown for (La_0.8_,Sr_0.2_)(Cr_0.98_,V_0.02_)_3_/CGO/NiO and (Sr_0.86_,Y_0.08_)TiO_3_/CGO/NiO anodes [248,249]. Recently, Cassidy *et al.* reported the integration of the LSCM anode in the Rolls-Royce IP-SOFC concept, the power density is relatively low with 75 mW/cm^2^ [250].

Martinez-Arias *et al.* showed the possible application of a cerium-terbium based anode for SOFC [251]. Tomita *et al.* described the RedOx stability of a BaCe_0.8_Y_0.2_O_3_ anode but the power density is very low [252]. 

Ca- and Co-doped yttrium chromite and samaria-doped ceria (SDC) composite anode in a ESC configuration was tested under multiple RedOx cycles at 800 °C without degradation due to its chemically and dimensionally stable behavior [253].

Strontium molybdate (SrMoO_4_)-YSZ composite with 1 vol % Pd catalyst appears stable after a RedOx cycle at 800 °C and gave a relatively good performance of 0.3 W/cm^2^ [254].

**Figure 34:**
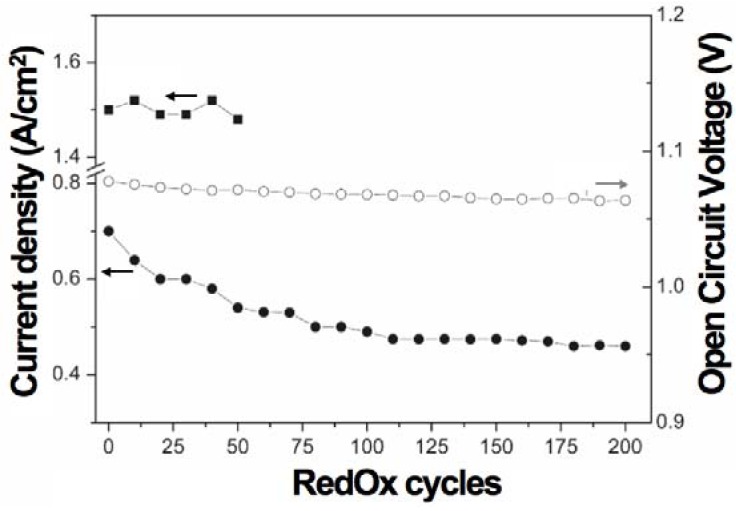
Open circuit voltage (open circle), current density at 0.7 V (10 min in H_2_ and 10 min in air) at 750 °C (closed circles), and current density at 800°C applying 2 h in H_2_ and 10 min in air (closed square), all as a function of the number of RedOx cycles with SYT ceramic anodes [260,261].

The Forschungszentrum Jülich has studied the doping of strontium titanate as SOFC anode for many years [15,234,241,255,256,257,258,259]. Recently, a breakthrough for a ceramic anode supported cell with high performance and RedOx stability was achieved with a Sr_0.895_Y_0.07_TiO_3_ (SYT) support, a (Sr_0.89_Y_0.07_)_0.91_TiO_2.91_-YSZ anode impregnated with 3 wt % NiO, and the YSZ electrolyte protected with a thin 20GDC interlayer so as not to react with a LSC cathode [260,261]. A current density of 1.5 A/cm^2^ at 800 °C and 0.7 V was obtained. The OCV decreased only by 1% over 200 RedOx cycles (of 10 min in H_2_ and 10 min in air) at 750 °C, whereas the current density lowered by about 40% during the 200 RedOx cycles (see Figure 34) [260,261]. A more recent study showed that the same cell tested in [260,261] had an OCV decrease of 5% after two RedOx cycles of 5 h under air at 750 °C (compared to the previous cycles of only 10 min under air). Interestingly, the performance reactivated after these cycles, which could be due to hot spots at the thin electrolyte cracks [262].

#### 3.5.3. Mechanically Stronger Materials

By increasing the fracture strength of mechanical support materials, fewer cracks will appear after a RedOx cycle. Klemensø *et al.* used this approach to find a more RedOx stable anode-supported cell [26,28]. They used 3YSZ in the anode support instead of 8YSZ as the former’s bending strength is four times higher than the latter’s. The addition of about 1 wt % of Al_2_O_3_ further enhanced the strength of the support. 

The addition of YSZ or high strength material fiber can increase the strength of the anode support. The fibers should be co-fired with smaller YSZ particles. 

#### 3.5.4. Use Support with Higher Thermal Expansion Coefficient (TEC)

Robert *et al.* observed better RedOx stability with higher NiO content. A possible reason for the better stability is the larger electrolyte compression stress [185]. The maximal strain accepted from the electrolyte is 0.04% without residual stress of the electrolyte. By including a compressive residual stress of 240 MPa in the electrolyte after firing, the maximal strain increased to 0.17%, as described by Klemensø [28]. If the compressive stress reaches 440 MPa, the maximal strain can be improved at least to 0.3%. The electrolyte can be put artificially under compression using a higher TEC support. The important point will then be thermal cycling stability and the stacking of these cells that will present higher curvature. 

### 3.6. Kinetics

#### 3.6.1. Oxidation Barrier

Applying a nickel-rich layer on the anode support (opposite to the electrolyte) may stop oxygen diffusion to the anode. As the nickel-rich layer oxidizes first, the porosity closes and the oxygen diffuses slowly to the RedOx sensitive ALF. At 750 °C, it takes almost twice the time to reach the same degradation with the oxidation barrier compared to the standard cell [143,263]. This extra time could allow the cooling of the system to a safe temperature, preventing RedOx degradation. 

#### 3.6.2. Improved Sealing

The idea is to block fuel gases into the anode compartment with valves during stack cooling. This requires efficient sealing, like glass-ceramics, to prevent any leakage. Versa Power Systems tested their standard cell by closing the inlet and outlet of the anode during 15 h at 750 °C and no degradation was observed [143].

#### 3.6.3. Lower Operating Temperature

The decrease of operating temperature down to 700 °C will strongly reduce the reoxidation kinetics and maybe limit the RedOx problem. Using scandium-stabilized zirconia (ScSZ) for electrolyte will decrease the ohmic loss due to lower ionic conductivity of YSZ at low temperature [264]. Toho Gas built a 1 kW stack based on ScSZ electrolyte [265]. 

Ni-Fe anode substrate with La_0.73_Sr_0.1_Ga_0.64_Mg_0.26_O_3−d_ (LSGM) electrolyte and Sm_0.4_Sr_0.6_Co_1.6_O_3−d_ (SSC) cathode gave 0.16 W cm^−2^ at 673K with RedOx stability for 2 cycles (2 h under oxidizing atmosphere) [266]. Scale-up of this technology still remains to be achieved.

The main limitation at low temperature will come from the cathode activity for oxygen reduction.

## 4. Synthesis for Ni-Based Anode-Supported Cells

**Figure 35:**
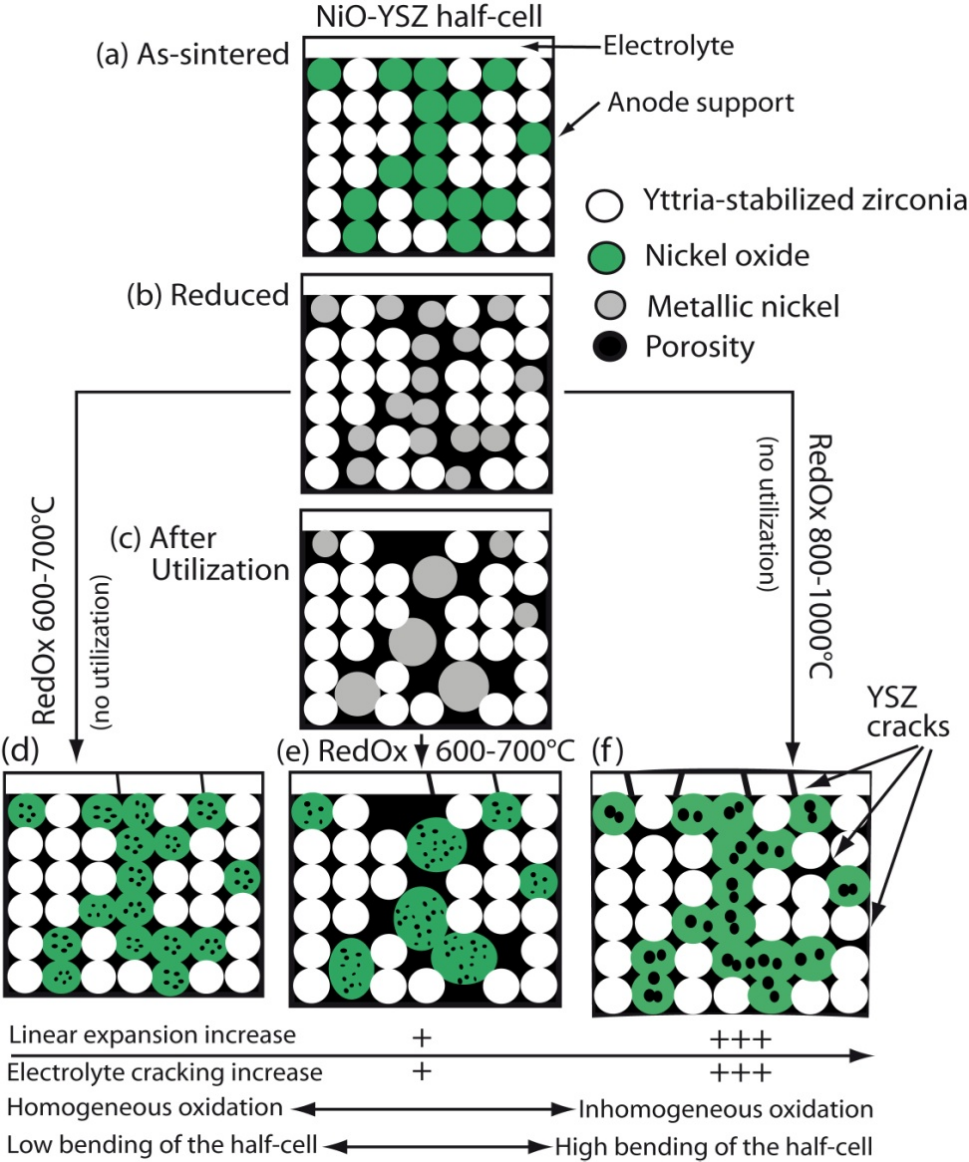
Scheme of RedOx instability of standard anode supported Ni-YSZ half-cell [129].

Based on the literature review of the RedOx instability of standard anode-supported cells given in Section 2, Figure 35 summarizes the major trends. In this scheme, the electrolyte is supported by the anode, where sintered grains are represented by circles. During the reduction, the porosity increases due to the volume reduction of about 41% of nickel oxide to metallic nickel. From this state, the standard half-cell can be subjected to three different treatments: (1) reoxidation at low temperature (600–700 °C, Figure 35d); (2) utilization of the half-cell with nickel coarsening (Figure 35c) and reoxidation at low temperature (Figure 35e) or (3) reoxidation at higher temperature (800–1000 °C, Figure 35f). 

At low temperature (case 1), nickel reoxidation is homogeneous through the whole anode support layer due to the faster gas diffusion compared to solid-state diffusion. The nickel oxide presents fine closed porosity created by the outward diffusion of Ni^2+^ through the nickel oxide grain boundaries during oxidation. Due to the closed porosity, the volume difference between reoxidized and as-sintered nickel oxide is positive. This induces a volume increase of the anode support and therefore tensile stresses and cracks in the thin electrolyte. 

During utilization (case 2), the nickel phase reorganizes to lower its surface energy. Nickel coarsening will be halted by the zirconia backbone after a few hundreds of hours. If reoxidation occurs after Ni coarsening, the net volume increase of nickel oxide should be similar to the one without utilization. But due to the reorganization of the nickel phase inside the composite, its volume increase could be higher because of the creation of porosity between the nickel oxide and zirconia phase. The higher linear expansion of the anode support after coarsening was confirmed by Pihlatie *et al.* [72]. The effect of temperature on the RedOx instability of Ni-YSZ anode-supported cells is more important compared to nickel coarsening. 

During oxidation at high temperature (800–1000 °C) (case 3) without previous utilization, the kinetics of solid-state diffusion is faster than gas diffusion, which induces an inhomogeneous oxidation and a sharp reduced/oxidized interface in the composite. This will develop high stresses and non-linear deformation inside the anode support. The composite creep creates bending of the cell towards the anode. First, at higher temperature, the porosity of the reoxidized nickel is coarser. The self-diffusion of nickel cation changes from shortcut-path-controlled to crystal-lattice-controlled between 700 and 1000 °C [47]. This can have an influence on the NiO internal porosity. Second, cracks in the YSZ backbone are observed and located at the zirconia grain boundaries due to higher diffusion at this location. These two effects make the linear expansion of the anode composite increase at high temperature. The higher volume expansion and the bending effect are cumulated and create higher crack density of the thin electrolyte. 

**Figure 36:**
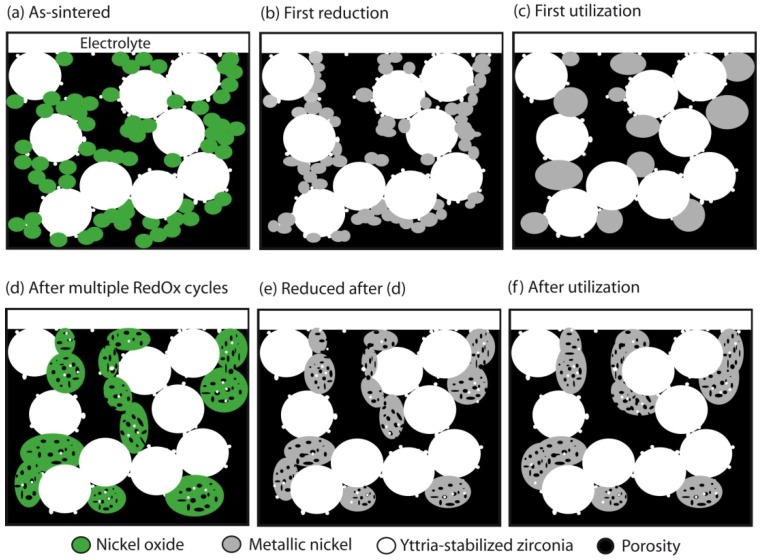
Scheme of RedOx behavior of highly porous Ni-YSZ anode supported half-cell [129].

As described in the general discussion, the Ni-YSZ anode-support microstructure can be modified to enhance its RedOx stability. The key measure is the porosity increase, to allow more space for the nickel oxide volume increase during RedOx cycles. This could be achieved by pore-former addition and using coarser zirconia particles. Figure 36 schematically depicts the behavior of an anode support optimized for RedOx stability during utilization and multiple RedOx cycles (as well as micrographs shown in Figure 28). An optimized microstructure includes fine nickel oxide to enhance the electrical conductivity, coarse zirconia to increase the porosity and a small addition of fine zirconia needed for sintering and stabilizing the microstructure. During first utilization, the performance and the electrical conductivity drop rapidly because of important coarsening of the conductive phase (Figure 36). The RedOx optimal microstructure has wider voids in the YSZ backbone, which means the nickel phase can reach coarser sizes. In addition, as the porosity is higher, the conductivity is lower. After multiple RedOx cycles, the electrolyte does not show cracks thanks to the low volume expansion of the highly porous support. A NiO internal porosity similar to that in the standard anode-microstructure is observed after RedOx cycles. But this internal porosity remains unchanged during reduction and during utilization due to fine zirconia particle encapsulation during the multiple RedOx cycles. The nickel volume is equal but, as it contains stable porosity and fine zirconia particles, its connectivity within the anode is increased compared to the microstructure after first utilization. 

High porosity decreases strength and conductivity [86,93,186,267]. Yet an anode support of optimal porosity and composition should retain sufficient mechanical properties, electrical conductivity and RedOx stability.

**Figure 37:**
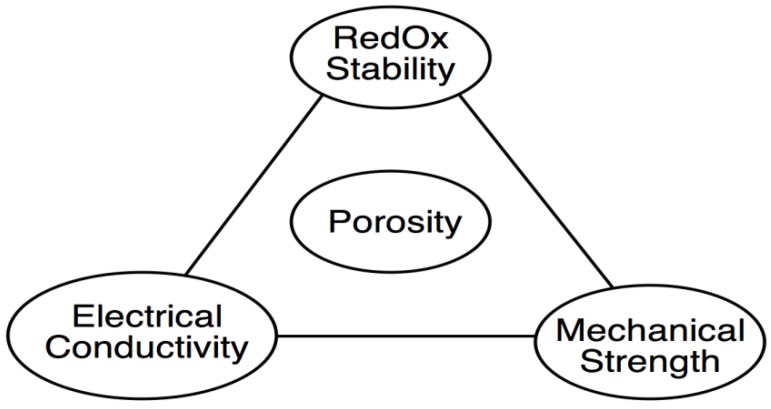
The central role of porosity in the Ni-YSZ anode supported solid oxide fuel cells properties.

As described through the different scales reviewed in this work (from nano to macrostructure), the key issue for RedOx stability is porosity. Optimal compositions of RedOx stable anodes contained between 47% and 52% of porosity [86]. On the other hand, porosity will decrease electrical conductivity and mechanical strength of the Ni-YSZ composite. Hence an optimal porosity should be determined to achieve a RedOx stable, highly conductive and strong anode support. Figure 37 depicts the central role of porosity in the Ni-YSZ anode support properties. The conductivity could be increased by adding more nickel and by changing the shape of the nickel (foam, coated pore-former or ceramics [201,202,204]). To enhance mechanical stability (maximal force at rupture), the thickness of the anode support should be increased. 

## 5. Conclusions

The key advantage of fuel cells is their high efficiency for converting chemical energy from a variety of fuels directly into electricity. 

Solid oxide fuel cells (SOFCs) are among the most interesting fuel cells technologies due to the highest efficiencies achievable even for small systems, to their ability for co-generation (heat and electricity) and to their feedability with many hydrocarbon-based fuels with manageable pretreatment or cleaning. Current SOFC limitations remain a certain fragility of the components, the high cost of some materials and the performance degradation at operating temperature.

Reduction and oxidation (RedOx cycle) of the Ni-YSZ anode at high temperature can decrease dramatically the performance of SOFC, especially for anode-supported cell designs. The volume increase during nickel oxidation induces tensile stress and cracks in the thin electrolyte. The irreversibility of the RedOx cycle is due to different causes:

(1) The internal porosity of NiO increases after reoxidation. The oxidation is governed by cationic diffusion: Outward diffusion of Ni^2+^ creates pseudo-Kirkendall porosity within the NiO particles.
(2) The nickel coarsening during anode utilization creates a rearrangement of the phase. During reoxidation NiO does not reoccupy its original sites. Water vapor presence increases coarsening of the nickel phase.
(3) Higher temperature (>700 °C) induces inhomogeneous oxidation (only the outer surface layer is oxidized) that produces bending of the cell. This can increase the stress in the thin electrolyte.
(4) Low partial pressure of oxygen and high water vapor pressure induce inhomogeneous oxidation similar to the one described under (3).

Solutions proposed in the literature are summarized in Figure 22. These solutions can be separated in two main families: (i) system and (ii) material solutions. To reach a RedOx stable system, the two families could be used in conjunction. System solutions suit large systems better while material solutions can be used for any system size. For the material solutions, the main routes are variations in: (a) stack design, (b) cell design, (c) materials choice, (d) Ni alloying, (e) kinetics of oxidation and (f) microstructure of the Ni-ceramic composite. All of these directions are potentially interesting. To reach a RedOx stable microstructure keeping a Ni-ceramic composite, the following aspects can be optimized:

(1) Porosity enhancement.
(2) Graded composition with more YSZ close to the electrolyte and the gas outlet to reach higher fuel utilization.
(3) Particle size and particle size ratio between NiO and YSZ. Coarse microstructures are more RedOx stable.
(4) Lower sintering temperature.
(5) Ni foam and Ni-coated pore-former and ceramic phase.
(6) Ni wet impregnation of the ceramic skeleton. The important drawback of this technique is the multiple impregnation and calcination cycles needed to reach even then only a few wt % of impregnated Ni.

All these microstructure changes to reach RedOx stability, especially the increase in porosity, should be considered in the light of other needs of the anode and the cell. The support and the anode composition can be modified. For the anode support, the optimal microstructure should have a good conductivity, a low expansion during RedOx cycles and a high strength. For the anode active layer, the optimal microstructure should possess a good electrochemical activity for fuel oxidation and a relative low expansion during RedOx cycles.

Finally, the optimal solution for RedOx instability of the solid oxide fuel cells anode will be a conjunction of different solutions at multiple levels of the SOFC module. Before building a SOFC system, the cost for RedOx instability solutions versus total cost should be evaluated. This will depend on the system size and its utilization mode. For large stationary modules, the system solution would be the first choice (including a secondary solution like cell-design, for example); for a small mobile module (like auxiliary power unit, APU), the solution is a combination of stack-design, cell-design, material choice and microstructure optimization. A RedOx stable system could for example be based on a good electrically conductive porous support (like doped SrTi-oxide or high temperature stainless steel) with a thin porous ceramic layer (like SmCe-oxide or doped YZr-oxide) impregnated with electrochemical active particles (like Ni and Ce-oxide). The dense thin electrolyte could be LaSrGaMg-oxide or standard stabilized-zirconia. A low temperature active cathode (like LaSrCoFe-oxide or SmSrCo-oxide) can be used, together with an interlayer against diffusion or reaction for long term stability. 

RedOx stability is still only one requirement of a good anode and anode-support. The optimal anode should have a coefficient of thermal expansion similar to other stack components, display high electrochemical activity for hydrogen, CO and hydrocarbon fuel oxidation and be chemically stable with respect to the other stack components. The stability over time (*i.e.*, no change in microstructure), with hydrocarbon fuels and with impurities like sulfur, phosphorus and others, is also essential. All these requirements should be fulfilled in order to obtain the optimal solid oxide fuel cells anode.

## List of Variables

*C_p,m_*: Molar heat capacity (J/mol K)
*d_V50_*: Mean particle size in volume (µm)
*D*: Diffusion coefficient (m^2^/s)
*δ*: Thickness of the NiO grain boundaries (m)
*E_a_*: Activation energy (kJ/mol)
*E*: Young modulus of the electrolyte (GPa)
*ε_ox_*: Oxidation strain (–)
*F*: Faraday constant = 96485 (A s/mol)
*F_u_*: Fuel utilization (%)
*G_ASC_*: Stored energy release rate (J/m^2^)
*G_c_*: Critical energy release rate (J/m^2^)
∆*G*: Gibbs free energy (J/mol)
*g*: the grain size (m)
*v*: Poisson ratio (–)
*h*: Electrolyte thickness (m)
*∆H*: Enthalpy (J/mol)
*i*: Current density (A/cm^2^)
*k*: Reaction rate (1/s)
*k_0_*: Reaction rate constant(1/s)
*M*: Molar mass (g/mol)
*p_O2_*: Partial pressure of O_2 _(–)
*R*: Gas constant = 8.314 (J/mol K)
*R_p_*: Polarization resistance (Ohm cm^2^)
*R_s_*: Ohmic resistance (Ohm cm^2^)
*ρ*: Density (g/cm^3^)
*T*: Temperature (°C or K)
*t*: Time (s)
*U*: Cell potential (V)
*V*: Molar volume (cm^3^/mol)
*x*: Degree of conversion (–)
*y*: Oxide layer thickness (m)

## List of Abbreviations

AFL: Anode functional layer
AS: Anode support
ASC: Anode supported cell
ASR: Area specific resistance (Ohm cm^2^)
CCL: Current collecting layer
CFD: Computational fluid dynamics
CGO: Ceria gadolinia oxide
CRS: Cumulative RedOx strain
CSC: Cathode supported cell
CTE: Coefficient of thermal expansion
DoE: Design of experiment
DoO: Degree of oxidation
EIT: Impulse excitation technique
EP: Electrochemical performances
ESC: Electrolyte supported cell
FEM: Finite element modeling
FIB: Focused ion beam
GDC: Gadolinia doped ceria
ISSC: Inert substrate supported cell
FZJ: Forschungszentrum Jülich
LSCF: LaSrCrFe-oxide
LSCM: LaSrCrMg-oxide
LSCV: LaSrCrV-oxide
LSGM: LaSrGaMg-oxide
LSM: LaSrMn-oxide
LST: LaSrTi-oxide
MSC: Metal supported cell
OCV: Open circuit voltage
RedOx cycle: reduction-oxidation cycle
RT: Room temperature
SRU: Single Repeating unit
ScSZ: Scandia stabilized zirconia
SDC: Samarium doped ceria
SEM: Scanning electron microscopy
SIS: Segmented-in-series
SOFC: Solid oxide fuel cell
SOEC: Solid oxide electrolyzer cell
SSC: SmSrCo-oxide
SYT: SrYTi-oxide
TEC: Thermal expansion coefficient
TEM: Transmission electron microscopy
TGA: Thermogravimetric analysis
TPB: Triple phase boundary
TPR: Temperature programmed reduction
TZP: Partially stabilized zirconia
WI: Wet impregnation
XRD: X-ray diffraction
YSZ: Yttria stalilized zirconia

## Appendix

**Table A1:** Summary of RedOx cycle effect on open circuit voltage (OCV) and electrochemical performance degradation (DEP).

Cell type	Anode and support materials	OCV %/Cy ^a^	EP %/Cycle ^b^	RedOx conditions	RedOx ^c^	Cell ^d^	Active Surf ^e^	Source reference
**Planar Design**								
Anode Supported Cell	Ni-YSZ	−56	–	850 °C, fuel gas off, 4 min	1	1	–	Cassidy *et al.* [10]
	SYT-YSZ AFL impregnated with 3 wt % NiO on SYT	~0	−0.2, *i* at 0.7 V	750 °C, air, 10 min	200	1	1	Ma *et al.* [261,262]
	SYT-YSZ AFL impregnated with 3 wt % NiO on SYT	−2	+14.6, *i* at 0.6 V	750 °C, air, 5 h	2	1	1	Ma *et al.* [261,262]
	Ni-YSZ AS	0	−2.3, *i* at 0.6 V+4.5, ASR 0.65–0.85 V	800 °C, full RedOx cycle, flowing air	11	1	1	Faes *et al.* [186]
	Ni-YSZ AS	0	−2.5, *i* at 0.6 V	800 °C, full RedOx cycle, flowing air	12	1	1	Faes *et al.* [187]
	Ni-YSZ AS	−0.1	+1.75, *i* at 0.75 V	800 °C, full RedOx cycle, fuel gas off	40	1 SRU	48	Faes *et al.* [187]
	Ni-YSZ AFL on Ni-YSZ AS	−0.75	−1.48, *U* at 0.75 A/cm^2^	750 °C, blowing air at 0.12 l/min for 20, 40, 60, 120, 240 and 360 min.	6	1 SRU	80	Waldbilig *et al.* [124]
	Ni-YSZ AFL on Ni-YSZ AS	–	−0.08, *U* at 0.75 A/cm^2^	750 °C, steam purge on Ni bed.	32	1 SRU	80	Wood *et al.* [144]
	Ni-YSZ AFL on Ni-YSZ AS	–	0, *U* at 0.5 A/cm^2^	750 °C, fuel gas off valves closed, 15 h.	1	1 SRU	80	Wood *et al.* [144]
	Ni-YSZ AFL on graded Ni-YSZ AS	−0.18	−0.38, *U* at 0.25 A/cm^2^	800 °C, full RedOx cycle	16	1 SRU	100	Ihringer [215]
	Ni-YSZ AFL on Ni-YSZ AS	+0.12	+2.1, *U* at 0.25 A/cm^2^	Via ionic current under N_2_,DoO = 0.6%, *i* = 0.25 A/cm^2^	17	1	1	Hatae *et al.* [121]
	Ni-YSZ AFL on Ni-YSZ AS	−1.0	+13, *U* at 0.25 A/cm^2^	Via ionic current under N_2_, DoO = 31%, *i* = 0.25 A/cm^2^	2	1	1	Hatae *et al.* [121]
Electrolyte Supported Cell	Ni-YSZ on 8YSZ	–	+6, *R_p_* at 950 °C	950 °C, fuel gas off, 20 min	3	1	10	Fouquet *et al.* [66]
	Ni-YSZ on 3Y-TZP	0	+12.5, *R_p_* anode−3.9, TPB length	1000 °C, O_2_ flowing, 10 min	4	1	0.28	Sumi *et al.* [138,139]
	Ni-CGO	0	+0.8, ASR 0.9–0.75 V	950 °C and 850 °C, air during 1 h	50	1	1	Sfeir *et al.* [163]
	Ni-8YSZ on 10Sc1CeSZ	–	+0.34, ASR at 0.71 A/cm^2^	850 °C, fuel gas off for 3 h	10	1	16	Glauche *et al*. [165].
	Ni-GDC on 3Y-TZP	–	−0.125, *i* at 0.7 V	850 °C, fuel gas off, full oxidation	100	1	16	Ouweltjes *et al.* [167]
	Ni-8YSZ: 30–50 µmNi-GDC: 40 µmNi-GDC: 30 µm	000	−0.9, *i* at 0.7 V−0.55, *i* at 0.7 V−0.15, *i* at 0.7 V	800 °C, flowing air, 1 min first 50 cycles and 10 min last 50 cycles	100	1	16	Ettler *et al*. [14]
	40GDC on 8YSZ	0	0, no graph shown	1000 °C, fuel gas off, until OCV = 0 V	several	1	10	Marina *et al.* [236]
	LSCM-GDC infil. with NiO on GDC	–	+2.2, *U* at 0.3 A/cm^2^	750 °C, 30 min under air	4	1	0.12	Barnett *et al*. [248]
	LSCV-GDC infil. with NiO on GDC	–	−0.6, *i* at 0.5 V	30 min under air, 30 min. 6.7% H_2_ in Ar	5	1	0.12	Barnett *et al*. [249]
	LSCV-GDC infil. with NiO on GDC	–	0, *U* at 0.2 A/cm^2^	30 min under air, 30 min under propane	5	1	0.12	Barnett *et al*. [249]
	Porous YSZ impregnated with Ni on 8YSZ	–	+5.8, *i* at short circuit (0 V)	800 °C, air, 15 min	15	1	–	Buyukaksoy *et al.* [210]
	Ni-CGO	0	+2.2, ASR 0.9–0.75 V	900 °C, air during 10 h	11	5 SRU	100	Sfeir *et al* [163]
	Ni-CGO on ScSZ	–	+2.3, ASR 0.9–0.75 V	900 °C, fuel gas off until full oxidation	15	5 SRU	100	Mai *et al.* [164]
	Graded Ni-20SDC on stabilized zirconia	–	+1, *R_p_* anode−3.2, *R_s_* anode	850 °C, fuel gas decrease to zero, driving current	1	5 SRU	–	Batawi *et al.* [132]
	Ni-CGO on 3Y-TZP	–	−0.14, *U* at 0.188 A/cm^2^	850 °C, fuel gas off and cool down in 4 h	83	30 SRU	127.8	Brabandt *et al.* [166]
	Ni-CGO on 3Y-TZP	–	−1.0, *U* at 0.172 A/cm^2^	800 °C, air flushing at 10 l/min for 20 min	3	30 SRU	127.8	Brabandt *et al.* [166]
Metal Supported Cell	Ni-YSZ (plasma) on CroFer22APU	0	−0.13, *U* at 0.3 A/cm^2^	800 °C, full RedOx cycle	20	1	12.5	Szabo *et al.* [174]
	Ni-YSZ (plasma) on CroFer22APU	0	−0.45, *U* at 0.25 A/cm^2^	800 °C, pure O_2_, 1 h	20	2 SRU	82	Szabo *et al.* [133]
	ScYSZ-infiltrated with 20GDC and 10 wt % NiO on CrFe 350	−0.1	−0.01, *i* at 0.7 V	800 °C, air, 1 min (first 50 cycles) and 10 min (last 50)	50 + 50	–	–	Blennow *et al.* [175]
CSC	10 μm 50:50 Pd:YSZ, 10 μm 8YSZ, 1 mm LSM	0	+3.3, *U* at 0.05 A/cm^2^	800 °C, full RedOx cycle	2	1	8.1	Huang *et al.* [160]
ICSS	SIS Ni-YSZ and Ni-SDC on Sr_0.8_La_0.2_TiO_3_	–	0, *U* at 0.9 A/cm^2^	800 °C, flowing air for 30 min	7	1	0.5	Pillai *et al.* [180]
**Tubular Design**								
Anode Supported Cell	Ni-YSZ, ,Ø = 2 mm, *h* = 200 µm	–	−35, *i* at 0.5 V	600 °C, flowing air 4 h and 30 min	1	1	3.8	Dikwal [20].
	Ni-YSZ, ,Ø = 2 mm, *h* = 200 µm	–	−0.38, *i* at 0.5 V	600 °C, flowing air 5 min	52	1	3.8	Dikwal [20].
	Ni-YSZ, ,Ø = 2 mm, *h* = 200 µm	–	−72, *i* at 0.5 V	800 °C, flowing air 30 min	1	1	3.8	Dikwal [20].
	Ni-YSZ, ,Ø = 2 mm, *h* = 200 µm	–	−0.44, *i* at 0.5 V	800 °C, flowing air 5 min	52	1	3.8	Dikwal [20].
**Mixed Design**								
ISSC	SIS Ni-YSZ on MgO-NiO	0	−0.1, *U* at 0.24 A/cm^2^	775 °C-RT, start-stop without fuel gas	20	1 SRU	–	Fujita *et al.* [178]
	SIS Ni-YSZ on TZP	–	0, *U* at 0.9 A/cm^2^	800 °C, flowing air for 40 min	17	1 SRU	14.4	Pillai *et al.* [179]
**Single Chamber**								
ASC	YSZ impregnated with 35 wt % LSCF and 5 wt % Ni	–	0, *U* at 0.5 A/cm^2^	800 °C, flow O_2_/N_2_ (60/200 sccm ^f^), 15 min	11	1	–	Zhu *et al.* [209]

^a^ Variation of open circuit voltage per RedOx cycle in [%/cycle]; ^b^ Variation of electrochemical performance (DEP) per RedOx cycle [%/cycle] and the type of electrochemical properties. Degradation of EP occurs when ASR, *R_s_* and *R_p_* are increasing and when *U*,*i* and TPB are decreasing; ^c^ Number of RedOx cycle done on the cells; ^d^ Number of cells in the test, SRU is the repeating unit of a pre-commercial design; ^e^ Active surface of one cell in [cm^2^]; ^f ^sccm: Standard cubic centimeter per minute (0 °C, 1 atm).
